# Climate Change Challenges Grey Wolf Resilience: Insights From Dental Microwear

**DOI:** 10.1111/ele.70337

**Published:** 2026-02-11

**Authors:** Amanda A. Burtt, Neil F. Adams, Sabina Nowak, Robert W. Mysłajek, Michal Figura, Mark A. Purnell, Angela L. Lamb, Danielle C. Schreve

**Affiliations:** ^1^ School of Geographical Sciences University of Bristol Bristol UK; ^2^ Natural History Museum London UK; ^3^ Centre for Palaeobiology and Biosphere Evolution, School of Geography, Geology and the Environment University of Leicester Leicester UK; ^4^ Faculty of Biology, Institute of Ecology, Department of Animal Ecology and Evolution University of Warsaw Warszawa Poland; ^5^ British Geological Survey Nottingham UK

**Keywords:** adaptive strategies, climate change, dental microwear texture analysis (DMTA), dietary flexibility, Europe: Britain, Grey wolf ( *Canis lupus* ), interglacial, palaeodietary reconstruction, palaeoenvironmental change, Poland

## Abstract

The grey wolf exemplifies ecological resilience, having survived major climatic fluctuations since the Middle Pleistocene. Once the world's most widely distributed mammal, its range has been drastically reduced by human‐driven habitat loss, persecution and competition for resources. Although listed as of Least Concern globally by the IUCN, the omission of climate change as a threat raises critical questions about its future persistence. This study examines dietary flexibility in European grey wolves ( *Canis lupus* ) using dental microwear texture analysis (DMTA). We compare British Pleistocene wolves from the Last Interglacial (MIS 5e) and the penultimate interglacial (MIS 7a–c) and contemporary wolves from Poland. Results suggest that during periods of elevated global temperatures, wolves exhibit evidence of increased durophagy. These data demonstrate deep‐time dietary plasticity and recurrent behavioural shifts, indicating that while the grey wolf is resilient, future warming winters may significantly reshape wolf diets in the mid‐latitude ecosystems.

## Graphical abstract

We investigate dietary plasticity in European grey wolves across interglacial periods using dental microwear texture analysis. Enhanced durophagy is associated with warm climates in both modern Polish and British Pleistocene wolves, indicating deep‐time behavioural flexibility. These results suggest that future winter warming may significantly reshape wolf diets despite the species' long‐term resilience.

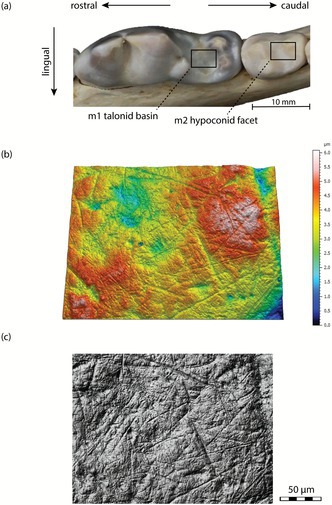

## 1 Introduction

The grey wolf ( *Canis lupus* L., 1758) is an ecological success story across the Holarctic, having persisted through multiple climate fluctuations since the Middle Pleistocene (Iurino et al. 2022). Once the world's most widely distributed mammal, its range has declined by about one third due to human persecution, including retaliation for livestock depredation and efforts to limit predation on wild game, as well as habitat fragmentation (Boitani et al. 2023). While the IUCN classifies the grey wolf's conservation status as being of Least Concern globally, the impacts of climate change are surprisingly not identified as one of the threats affecting this species (Boitani et al. 2023). Climate change, now considered the third greatest threat to mammals after overexploitation and habitat loss (Ripple et al. 2025), may paradoxically expand dietary opportunities for wolves in some regions even as their suitable habitats shrink (Helman et al. 2022). The species' high mobility may support adaptation in certain environments, though this capacity is unlikely to mitigate the species from climate‐related changes overall (Suel et al. 2018).

The ecological resilience of extant large carnivores can be understood through the adaptations of their past relatives to climate change, notably through the glacial–interglacial cycles of the Pleistocene. A particularly informative line of evidence for current species management and conservation comes from multiproxy palaeodietary reconstruction (e.g., Rivals et al. 2022). With respect to grey wolves, previous studies using morphometrics and dietary stable isotopes have highlighted their ability to respond flexibly to changing climatic conditions, in terms of targeting different prey species (Flower et al. 2021; Flower and Schreve 2014; Landry et al. 2021; Leonard et al. 2007). In addition, morphometrical studies have identified evolutionary trends in the grey wolf cranio‐dental apparatus that are consistent with adaptations toward consumption of flesh and non‐flesh items at different times in the past (Flower et al. 2021). However, while grey wolves have been highly successful in flexing their diet in response to past environmental change, a critical question remains as to whether this ability is sufficient to consider the species of minimal conservation concern in the future.

As a keystone species, the grey wolf has been a fundamental component of the Palaearctic ecozone for over 300,000 years, and today this species exerts a positive influence on biodiversity and trophic dynamics through a range of direct and indirect controls on ecosystem structure (McLaren and Peterson 1994; Ripple and Beschta 2012; Ripple and Larsen 2000). Carnivore prey choice is driven by individual ecological niche and resource partitioning (Wilmers, Crabtree, et al. 2003). Trophic interactions and herbivore numbers are driven by bottom‐up (i.e., resource limitation) and/or top‐down (i.e., predation) regulation (Crabtree and Sheldon 1999; Wilmers, Stahler, et al. 2003). In Europe, roe deer ( *Capreolus capreolus* ), red deer ( *Cervus elaphus* ) and wild boar ( *Sus scrofa* ) are most frequently targeted as wolf prey, along with reindeer ( *Rangifer tarandus* ) and elk ( *Alces alces* ) at high latitudes (Baranowska et al. 2025; Kojola et al. 2004; Nowak et al. 2005, 2011; Wagner et al. 2012). However, wolves adopt a more generalist diet when the availability of wild ungulates is seasonally or permanently low, hunting smaller mammals such as Eurasian beaver ( *Castor fiber* ) and brown hare ( *Lepus europaeus* ), as well as fish (Jędrzejewski et al. 2012; Mysłajek et al. 2021; Nowak et al. 2024; Stanek et al. 2017) or even consuming non‐meat foods such as berries (Homkes et al. 2020). Nevertheless, as highlighted by the International Union for Conservation of Nature, one of the most important questions still remaining about wolves involves the nature of their interaction with prey populations (Boitani et al. 2018), that is to say, how and why are dietary choices made and how do they vary through time?

**FIGURE 1:**
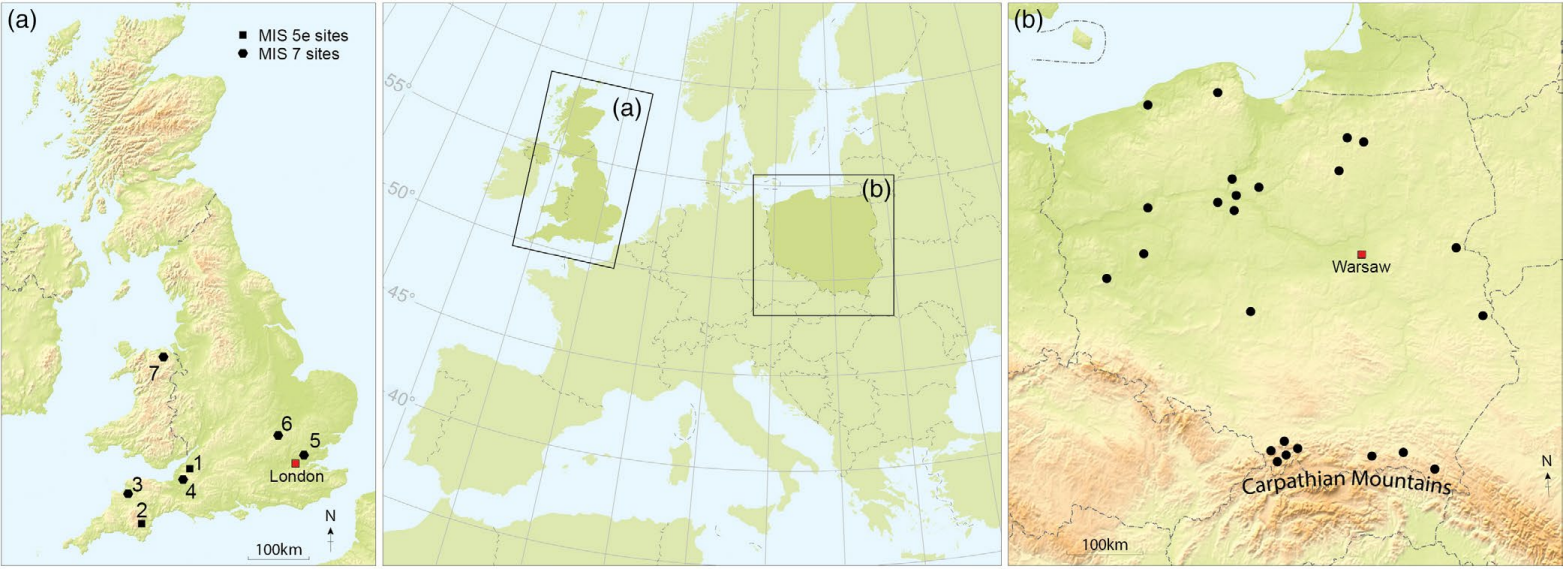
Sampling sites for fossil (A) and modern (B) wolves. (A) Fossil sites in Britain are MIS 5e, (1) Durdham Down Bone Cave, (2) Joint Mitnor Cave; Fossil sites in Britain are MIS 7a–c, (3) Bleadon Cave, (4) Hutton Cave, (5) Ilford, (6) Marsworth and (7) Pontnewydd Cave, St Asaph; (B) Poland sites for modern wolves. Base map from Maps in Minutes 2009, published by RH Publications.

This study examines the dietary adaptability of European grey wolves using dental microwear texture analysis (DMTA). Dental microwear refers to the microscopic scratches, pits and other wear patterns observed on the enamel surface of teeth (Walker et al. 1978). Dental microwear texture studies have established a robust link between tooth microwear features and dietary habits (e.g., DeSantis 2016; Hillson 2005; Scott et al. 2006; Teaford and Walker 1984; Ungar et al. 2008; Ungar et al. 2010; Walker et al. 1978). We compare modern Polish wolf populations from the present interglacial (Holocene), with fossil specimens from two contrasting Pleistocene interglacials: the warmer Last Interglacial (Marine Oxygen Isotope Stage [MIS] 5e) and the cooler Penultimate Interglacial (MIS 7a–c) (Figure 1). Wolves were extirpated from the British Isles centuries ago; therefore, Poland, with its permanent wolf population, serves as a suitable reference group against which interpretations of wolf dietary behaviour during past interglacials in Europe at similar latitudes can be ground‐truthed.

The two interglacial assemblages from Britain studied here reflect different palaeoclimatic and palaeoenvironmental conditions. The Last Interglacial (MIS 5e) was a period of enhanced global warmth (Lunt et al. 2013; Masson‐Delmotte et al. 2006) and a wide range of thermophilous flora and fauna were present that are common in southern Europe today (Candy et al. 2010). Palaeotemperature reconstructions in Britain (Coope 2010) reveal mean warmest month (July) temperatures 3°C to 5°C above present (taken to be +16°C to +17°C across most of Britain). Winters during the Last Interglacial were mild, estimated to be the same or slightly lower (Coope 2010; Gao et al. 2000) than the modern‐day coldest month (January), currently +3°C to +4°C (Candy et al. 2016). The presence in Britain of species intolerant of prolonged winter cold or frozen water bodies (Candy et al. 2016) indicates that seasonal snow cover was rare. Vegetation during the Last Interglacial is typified by thermophilous trees and shrubs, such as *Quercus* and *Corylus*, species typical of closed woodland, underlining the density of the forest at this time (Gao et al. 2000). Available prey for wolves included not only megaherbivores, such as straight‐tusked elephant (*Palaeoloxodon antiquus*), narrow‐nosed rhinoceros (*Stephanorhinus hemitochus*) and hippopotamus ( *Hippopotamus amphibius* ) but also wild boar, red deer, fallow deer ( *Dama dama* ), giant deer (*Megaloceros giganteus*), aurochs ( *Bos primigenius* ) and steppe bison (*Bison priscus*) (Currant and Jacobi 2011).

In contrast, the climatic pattern of the penultimate (MIS 7a–c) interglacial coincides with the most extreme peak in orbital eccentricity seen over the last 800 ka (Berger and Loutre 2002), producing a relatively “cool” temperate episode in comparison to MIS 5e (Berger et al. 2015; Candy et al. 2010; EPICA community members 2004; Ruddiman et al. 1989). Palaeotemperature reconstructions indicate a mean July temperature of +15°C to +16°C (comparable to, or marginally cooler than England today) and winter temperatures between 0°C and −5°C (e.g., De Rouffignac et al. 1995; Murton et al. 2001), allowing for the seasonal presence of snow cover on the landscape. Pollen spectra from MIS 7a–c sites demonstrate a strong predominance of non‐arboreal taxa, particularly grasses, sedges and dry ground herbs (Buckingham et al. 1996; Green et al. 1984; Murton et al. 2001), denoting the prevalence of open grassland habitats. Low percentages of arboreal pollen suggest patches of open woodland, rather than closed forest (e.g., Buckingham et al. 1996). Herbivore prey available for wolves was similar to that of MIS 5e, including straight‐tusked elephant, narrow‐nosed rhinoceros, red deer, giant deer, wild boar, aurochs, steppe bison, as well as steppe mammoth (*Mammuthus trogontherii*), horse ( *Equus ferus* ), steppe ass (*Equus hydruntinus*), Merck's rhinoceros (*Stephanorhinus kirchbergensis*) and woolly rhinoceros (*Coelodonta antiquitatis*) (Schreve 2019).

Between them, the seven Pleistocene assemblages cover a range of depositional environments (upland and lowland, cave and ‘open’ sites; Table S1) and contain many thousand specimens, thereby mitigating against any local taphonomic factors and providing a robust basis for inter‐comparison. Although competitor densities cannot be reconstructed in the past, the large carnivore guild was also similar across both Pleistocene periods (Schreve 2019). Dietary differences are therefore unlikely to reflect altered interspecific competition. The body mass of brown bear ( *Ursus arctos* ) was larger in MIS 5e (325–420 kg), compared to MIS 7a–c (270–310 kg), body masses of both spotted hyaena ( *Crocuta crocuta* ) and cave lion (*Panthera spelaea*) were comparable or smaller in MIS 5e than MIS 7a–c, at 65–71 kg and 185–205 kg, respectively, against 69 kg and 210–340 kg (Collinge 2001). Mean body mass of wolves remained comparable in both interglacials, at 33.54 ± 2.70 kg for MIS 5e and 34.03 ± 1.73 kg for 7a–c (Flower and Schreve 2014; Flower 2016). Given the ecological flexibility of wolves (Flower et al. 2021; Landry et al. 2021), it is improbable that access to large prey was restricted.

With respect to the modern dataset, Poland is considered a good comparator for Britain, being a lowland country with most terrain only a few hundred metres above sea level and located at a similar latitude (Figure 1). The Carpathian Mountain range, in the southern and southeastern part of the country, constitutes the primary area of high‐altitude terrain. Forest cover in Poland comprises almost 30% of the total land area, with western Poland exhibiting up to c.50% forest cover (https://dbw.stat.gov.pl). The climate is temperate, with both continental and maritime influences, and annual mean air temperatures between 1999 and 2018 of 8.9°C, with a gradient of higher temperatures in western Poland and lower temperatures in the east (Kubiak‐Wójcicka and Machula 2020). Current average winter temperatures are approximately 1.6°C, with higher elevations experiencing the lowest winter temperatures (Tomczyk et al. 2021). The native wild ungulate community and prey for wolves in Poland is abundant and consists of roe deer (most numerous), red deer, wild boar and elk (Borowik et al. 2013; State Forests National Forest Holding 2024; Niedziałkowska et al. 2024). Locally, and in much smaller numbers, European bison ( *Bison bonasus* ) occur. Among non‐native species introduced for hunting purposes, the most numerous is the fallow deer, while locally, species include sika deer ( *Cervus nippon* ) and mouflon ( *Ovis ammon* ). Most wild ungulate populations are increasing in number and range, especially those living in the mosaic of forests and farmlands, where they find favourable habitats and a rich food base (Ferens et al. 2025). The sole exception relates to the recent culling of wild boar to control African Swine Fever (State Forests National Forest Holding 2024; Morelle et al. 2020), although wild boar carcasses remain available for scavengers, including wolves. Domestic livestock form only a very marginal component of the diet, even when readily available (Baranowska et al. 2025; Nowak et al. 2005).

We test the null hypothesis that dental microwear textures do not differ significantly between MIS 5e and MIS 7a–c wolves. Alternatively, significant differences would provide evidence of dietary niche shifts, potentially associated with differences in climate between the two interglacials (i.e., warmer conditions of MIS 5e). Such a pattern would align with behavioural studies demonstrating that wolves adjust foraging strategies in response to climatic fluctuations (Sidorovich et al. 2017). Modern Polish wolves are included as a comparative reference representing dietary behaviour under warm interglacial–like conditions and provide a baseline against which fossil interglacial wolf diets can be evaluated.

We hypothesise that warmer interglacial conditions promoted more durophagous behaviour, likely through increased bone consumption from greater carcass utilisation. This hypothesis is consistent with DMTA evidence from modern North American wolves, in which recent warmer winters have been suggested to correspond with comparable dietary shifts (Burtt and DeSantis 2022). During MIS 7a–c, under cooler interglacial conditions, we predict that wolves experienced less dietary pressure and would be able to access a greater amount of flesh (i.e., less hard) resources. This trend is reported in modern wolves when winter temperatures are sustained for longer periods of time (Mech and Peterson 2003). If our null hypothesis is rejected and our alternative hypotheses find support, the data from Pleistocene wolves can be used to test whether modern wolves are under dietary stress in a currently warming world. These data provide insight into how grey wolves coped with past climate fluctuations in the absence of human influence. They also help identify subtle ecological stresses in modern populations that may signal vulnerability under ongoing and future warming.

## 2 Materials and Methods

**FIGURE 2:**
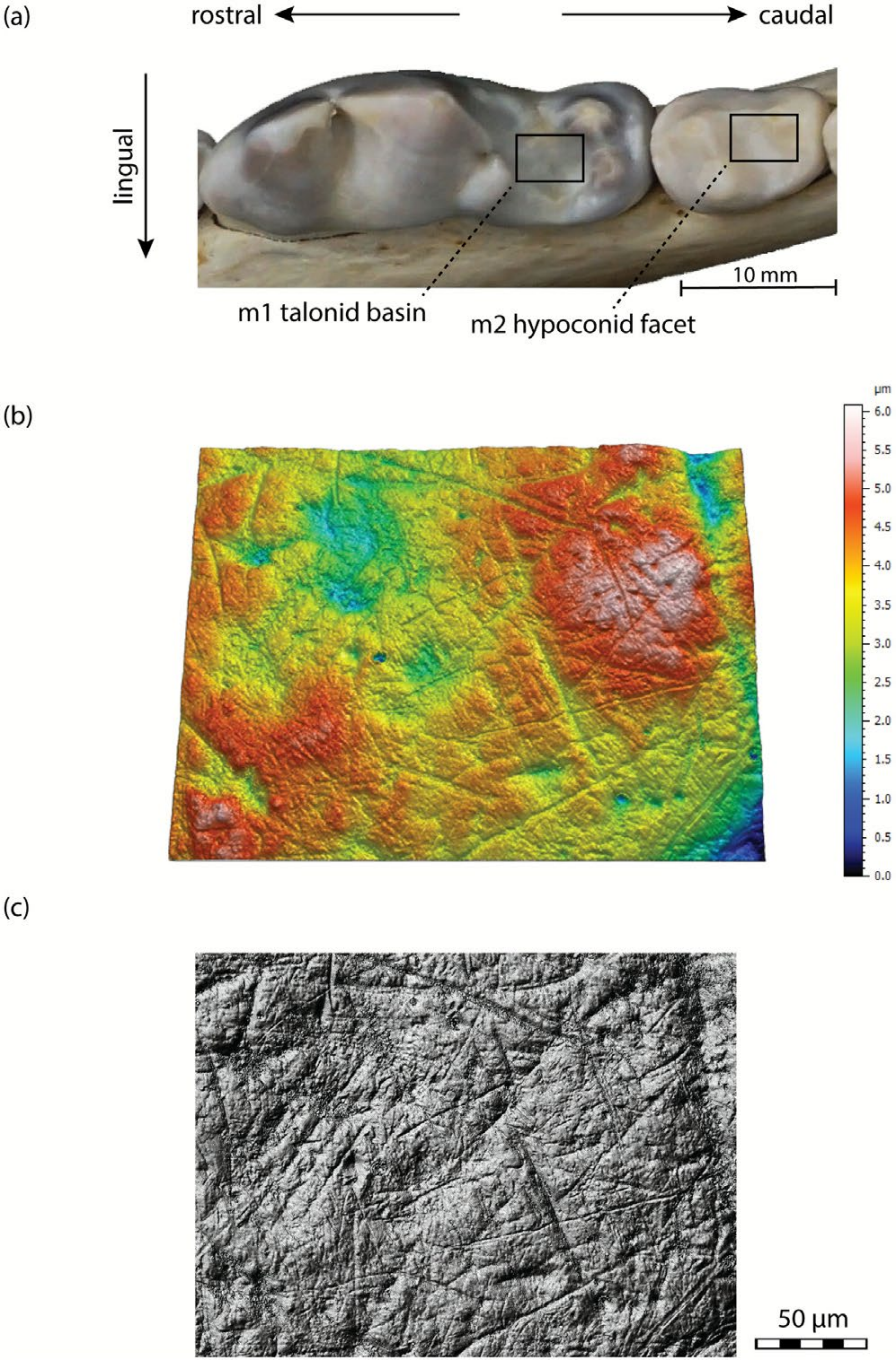
(a) Mandibular molars showing area of analysis, the talonid basin of the first mandibular molar and the hypoconid facet of the second mandibular molar (b) Rendered scans of wolf tooth surfaces with topographic contouring (c) Greyscale photo simulation of the same surface (Specimen Poland: F.2017.10.03).

Dental microwear textures were collected from first (m1) and second (m2) mandibular molars (Figure 2). Sixty‐two total wolf teeth (m1 and m2) with intact enamel and well‐preserved microwear surfaces were selected for analysis. Specimens exhibiting substantial enamel loss attributable to injury or advanced wear were excluded. The final sample comprises individuals from MIS 7a–c (m1, *n* = 7; m2, *n* = 7), MIS 5e (m1, *n* = 8; m2, *n* = 7), and modern wolves from Poland (m1, *n* = 11; m2 = 22). See Tables S1 and S2 for provenance information for all wolf specimens. In instances where data were collected from both the first and second mandibular molars of the same individual, priority was given to the data from the second molar. Total teeth used for diet reconstruction are MIS 7a–c: *n* = 13; MIS 5e: *n* = 13, and Poland: *n* = 22. Matched pair analysis, used to show correlation between first and second molars, included data from specimens with both molars.

This investigation examined the tooth enamel of grinding facets, specifically focusing on the anterolingual surface of the hypoconid cusp on lower second molars and the talonid basin of the first mandibular molar (see Figure S1). Post‐carnassial surfaces have been shown to be ideal for assessing bone consumption textures, serving as the accepted benchmark in microwear analyses of carnivorans. Data obtained from these surfaces allow for comparative analysis with other DMTA studies (DeSantis et al. 2015, 2019; Scott et al. 2005, 2006; Tanis et al. 2018; Ungar et al. 2010).

Teeth selected for this study were replicated by moulding the enamel surfaces and producing a cast. The dental specimen moulding method involves a non‐abrasive cleaning of the occlusal tooth surface using a liquid acetone/ethanol mixture. Subsequently, polyvinylsiloxane dental impression material (President The Original: Regular Body, Coltene‐Whaledent Corp., Altsatten, Switzerland) is applied. Post‐curing, the moulding agent is easily extracted. The cleaning and moulding process is limited to the occlusal surfaces of mandibular second molars and the talonid basin of the mandibular first molar. Tooth surface casts were then produced using high‐resolution epoxy (Epotek 301; Epoxy Technologies Corp., Billerica, MA, USA), which is optimised for imaging purposes. Epoxy tooth replicas were scanned with a Sensofar S neox profiler in the School of Geography, Geology and the Environment at the University of Leicester with the following settings: confocal scanning, 100× ELWD lens (numerical aperture 0.8), green LED light source (530 nm), z‐step size 0.07 μm, CCD 2448 × 2048 pixels, field of view 169 × 141 μm, spatial resolution (x,y) 0.07 μm. Four adjacent fields of view were scanned and cropped to an area of 242 × 181 μm. DMTA integrates surface profilometry, scale‐sensitive fractal analysis (SSFA), and areal surface texture analysis (based on a suite of parameters, most of which are defined by ISO‐25178‐2) to quantify dental surfaces in three dimensions. To note, the *NewEpLsar* SSFA variable has been introduced to replace the epLsar variable, thereby correcting a coding error in the original ToothFrax (Surfract Corp., Worcester, MA) software package (see Calandra et al. 2021).

MountainsMap Imaging Topography (version V10, Digital Surf, Besançon, France) was utilised to quantify dental microwear texture parameters from Sensofar scans. The scan processing protocol in MountainsMap comprises: (1) levelling with least squares plane (LSPL) to correct surface inclination, (2) threshold application to eliminate the upper and lower 0.1% of data, (3) filling of non‐measured point using the smooth shape calculated from neighbours function, (4) surface point retouching to manually edit dust and extraneous particles followed by filling of missing data points using the smooth shape calculated from neighbours function, (5) level again for generating SSFA parameters, (6) form removal with polynomial of degree – 2 (7) spatial filtering (robust Gaussian, order 0 (ISO 16610‐71); with a cut‐off value of 0.025 μm) for generating areal texture parameters.

We tested for differences between first and second molars when present in the same individual using matched pairs analyses with nonparametric paired Wilcoxon signed rank tests to test for significant differences between texture parameter values for data from m1 and m2 enamel surfaces. The Benjamini–Hochberg procedure was applied to control the false discovery rate (FDR = 0.05), and unless otherwise stated, reported differences reflect parameters that remain significant following application of this procedure. Many dental microwear texture parameters were not normally distributed (Shapiro–Wilk tests) and have unequal variances (Bartlett and Levene tests), even after log‐transformation. Nonparametric statistical tests were therefore used on untransformed raw data for analysis. Our hypotheses were tested using Wilcoxon two‐sample tests for comparisons between two groups, or Kruskal–Wallis tests between three groups. Nonparametric Steel‐Dwass pairwise tests were used following Kruskal–Wallis tests to test for significant differences between pairs of groups. Principal component analysis (on correlations; PCA) has been shown by previous work to be a powerful tool for analysis of multivariate DMT data across a range of fossil and extant fauna and dietary spectra (e.g., Bestwick et al. 2020; Gill et al. 2014; Purnell et al. 2012, 2013; Winkler et al. 2019). PCA was used to test whether Pleistocene (MIS 5e and MIS 7a–c) and modern wolf groups (Poland) occupied statistically different areas of multivariate texture‐dietary space. All statistical analyses were carried out in JMP Pro 18.2.1 Student Edition (JMP Statistical Discovery LLC, Cary, NC USA), except for the Benjamini‐Hochberg procedure, which used Microsoft Excel (McDonald 2014; http://www.biostathandbook.com/benjaminihochberg.xls).

## 3 Results

### 3.1 First and Second Molars

Nine modern Polish wolves had texture data from both m1 and m2 for comparison. No SSFA or areal texture parameters showed significant differences between m1s and m2s. We repeated the analyses after including three additional Pleistocene specimens for which both m1 and m2 data were available from the same individuals, and results were unchanged, with no parameters remaining significant following application of the Benjamini–Hochberg procedure (Tables S3–S6).

### 3.2 Pleistocene Wolves

**FIGURE 3:**
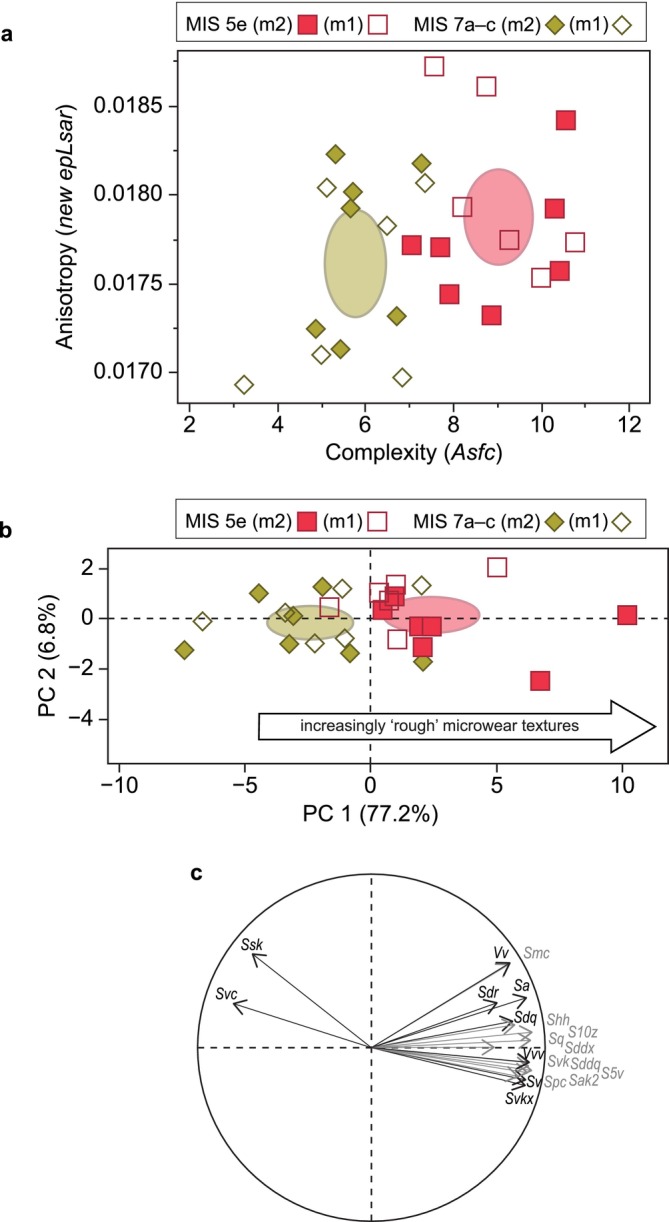
(a) Bivariate plot of (SSFA parameters) complexity and anisotropy, (b) PCA plot of 19 ISO parameters that significantly differ between MIS 5e and MIS 7a–c, (c) PCA loading plot, with vector length proportional to loading scores for each texture parameter on the PC axes. In (a) and (b) ellipses are 95% confidence limits for the group means.

**FIGURE 4:**
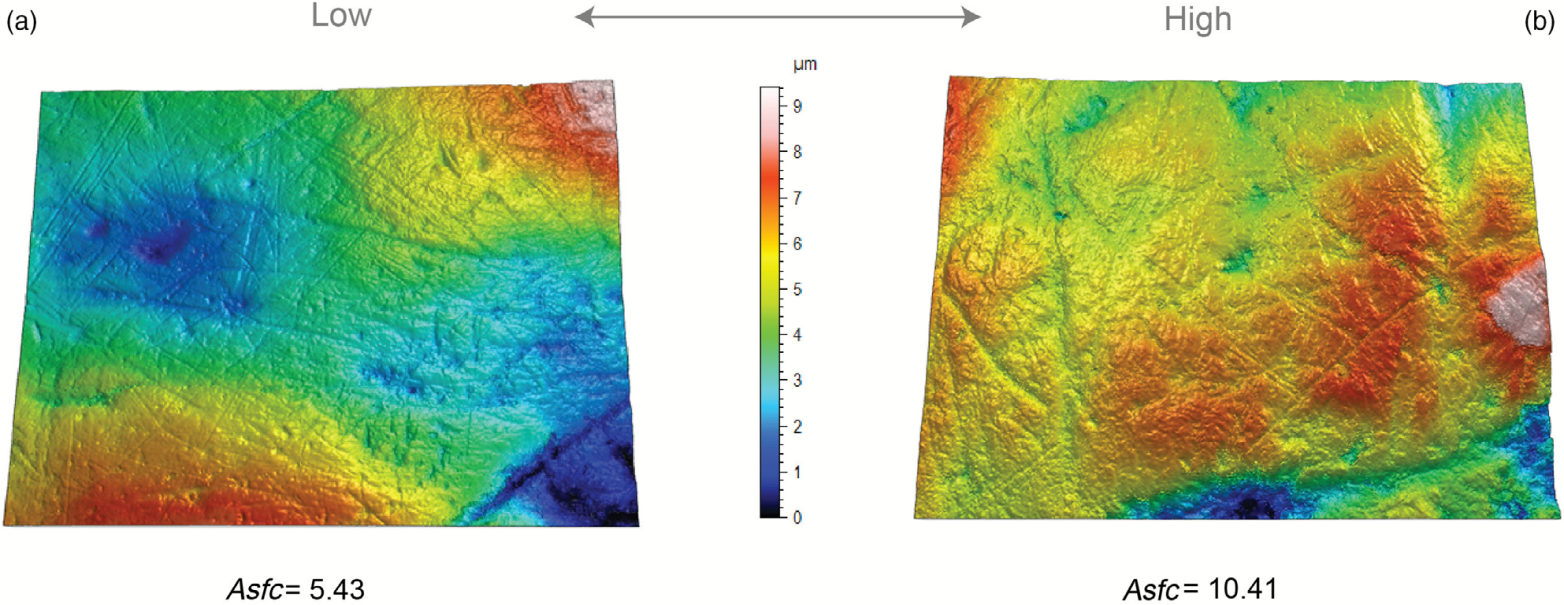
Rendered scans of wolf tooth surfaces with topographic contouring, illustrating a range of values for complexity (Asfc). (a) Low complexity value 5.43 (m2, British Geological Survey specimen Ilford UKBGS.GSM95739), (b) High complexity value 10.41 (m2, Torquay Museum specimen Joint Mitnor Cave TORMSP35085).

Several SSFA parameters show significant differences between wolves from the two temporal groups following correction for multiple comparisons (Table S7). Most of these parameters reflect differences in enamel complexity (e.g., Asfc), with MIS 5e wolves being significantly rougher than those from MIS 7a–c (*Asfc*: *Z* = 4.205, *p* < 0.0001; Figure 3a). Complexity variables are associated with bone processing, with higher values indicating greater hard food intake and carcass utilisation. These surfaces display a wide range of pit and scratch sizes and configurations, resulting in a roughened appearance (Figure 4). In contrast, anisotropy is linked to the consumption of tough foods such as flesh (Donohue et al. 2013; Schubert et al. 2010; Ungar et al. 2010), although no significant statistical differences were detected (*NewEpLsar Z* = 0.872, *p* = 0.383).

Nineteen areal texture parameters differ significantly between these temporal groups following Benjamini–Hochberg correction (Table S8). Principal component analysis confirms separation between wolves from MIS 7a–c and MIS 5e along PC 1 (*Z* = 3.282, *p* = 0.001), which explains 77.2% of variance (Figure 3b,c; Tables S9 and S10). Wolves from MIS 5e exhibit higher values in height, volume and feature parameters, related to surface peaks and valleys, as well as hybrid parameters associated with surface steepness, complexity and intricacy (see Section 4). Multivariate analyses of areal parameters indicate that MIS 5e wolves exhibit substantially more complex microwear textures than those from MIS 7a–c.

### 3.3 Comparison of Pleistocene and Modern Wolves

**FIGURE 5:**
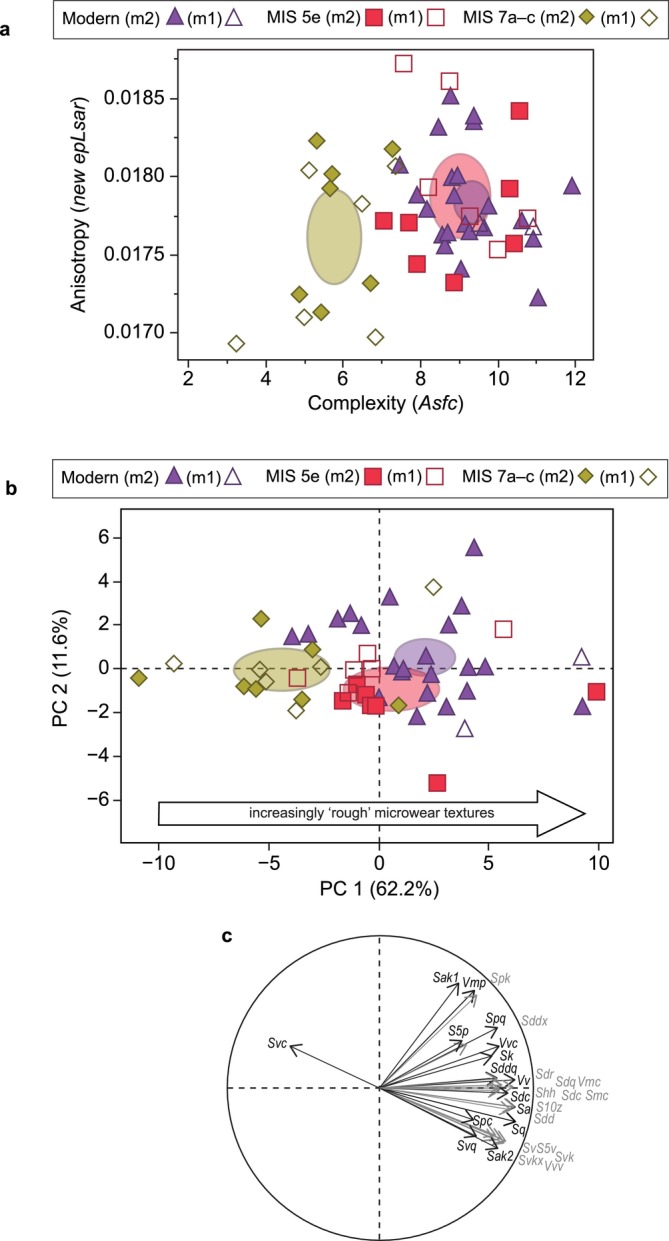
(a) Bivariate plot of (SSFA parameters) complexity and anisotropy for MIS 5e, MIS 7a–c, and modern Polish wolves. (b) PCA plot of the 30 ISO texture parameters that significantly differ between the three temporal groups. (c) PCA loading plot, with vector length proportional to loading scores for each texture parameter on the PC axes. In (a) and (b) ellipses are 95% confidence limits for the group means.

SSFA parameters also show significant differences in surface roughness between wolf dental textures from MIS 5e, MIS 7a–c and modern Polish wolves following correction for multiple comparisons (Tables S11 and S12). Complexity (*Asfc*) values differ between MIS 5e, MIS 7a–c and modern Polish wolves (Kruskal–Wallis test: *χ* ^2^ = 28.183, d.f. = 2, *p* < 0.0001). Pairwise comparisons reveal that wolves from MIS 5e do not exhibit significant differences in complexity from modern Polish wolves (Figure 5a; Table S13; Steel‐Dwass test: *Z* = 0.748, *p* = 0.735). In contrast, wolves from the penultimate interglacial (MIS 7a–c) exhibit significantly different complexity values from modern Polish wolves (Steel‐Dwass test: *Z* = 4.947 *p* < 0.0001). There are no significant differences in anisotropy values between the modern samples and those from either period in the Pleistocene.

Kruskal–Wallis tests reveal 30 areal roughness texture parameters differ significantly between the three temporal groups following Benjamini–Hochberg correction (Table S14). Principal component analysis shows separation between groups along the PC 1 axis (Kruskal–Wallis test: *χ* ^2^ = 19.649, d.f. = 2, *p* < 0.0001), which explains 62.2% of variance (Figure 5b,c; Tables S15 and S16). No such group separation is observed along PC 2 (*χ* ^2^ = 4.228, d.f. = 2, *p* = 0.121), which explains 11.6% of variance. Pairwise comparisons show that MIS 5e and modern wolves both have significantly greater PC 1 values than MIS 7a–c wolves (Steel‐Dwass pairwise tests: *p* = 0.005 and *p* = 0.0001, respectively), whereas MIS 5e and modern wolves do not differ significantly in PC 1 values (*p* = 0.181; Table S17).

## 4 Discussion

Based on our results, we reject the null hypothesis and suggest that British wolves from MIS 7a–c consumed significantly less hard food (i.e., rigid tissues) than either MIS 5e or modern Polish wolves, both of which we interpret as exhibiting evidence of greater durophagous behaviour. This pattern points to a dietary shift between interglacial periods.

In SSFA, both complexity and anisotropy are established parameters for dietary reconstruction, with durophagous behaviour typically interpreted from higher complexity values. Previous work has shown that complexity is effective at distinguishing between wolf groups, whereas anisotropy often remains indistinguishable, despite its broader use as a dietary benchmark (Burtt and DeSantis 2022; DeSantis et al. 2015). Consistent with this pattern, complexity measures in this study show marked differences in surface roughness, suggesting variation in durophagous behaviour, while anisotropy does not differ significantly between wolf groups.

There are many areal texture parameters that are linked to dietary behaviour in other vertebrates. For example, *Sq*, *Sdq*, *Vmc*, *Vvv*, *Svk* and *Sa* all had higher values in durophagous fish populations compared to populations that focus on vegetation with less hard‐shelled prey (Purnell and Darras 2016) and among reptiles, parameters including *Sa*, *Sq*, *Sdq*, *Smc*, *Vvv* and *Vmc* distinguished durophagous mollusc‐eaters from other taxa (Winkler et al. 2019). In mammals, *Sq*, *Sa*, *Vvv*, *Vmc* and *Svk* are positively correlated with dietary ‘hardness’ in bats (Purnell et al. 2013), whereas higher values of *Sa*, *Smc*, *Vmc*, *Vv* and *Vvv* occur in grazing mammals with highly abrasive diets compared to those of browsers (Schulz et al. 2013). Similarly, Kubo and Fujita (2021) found all the areal surface texture analysis parameters that strongly load PC 1 in our study (except *Sdd*, which was not included in their analysis) to correlate significantly and positively with the percentage of abrasive grass in the diet of extant Japanese sika deer ( *Cervus nippon* ). In primates, *S5v* and *Sq* were found to both be higher in species that consumed large hard particles (such as seeds, bark and insect cuticles) compared to species eating softer, more ductile leaves (Calandra et al. 2012). Most notably, dental textures comparing grey wolves from Sweden and Alaska (Schulz‐Kornas et al. 2024) supports our analysis. Swedish wolves feed on larger prey than those in Alaska and their diet is thought to include more bones. DMTA found significantly higher values of *S10z* and *Vvv* in the Swedish wolves as well as other areal texture parameters found to differ between wolf samples in our study, including *S5p*, *Sdr*, *Spc* and *Sv*. Sdd (‘mean dale local depth’). The only parameter with strong PC1 loadings not previously associated with diet, has been tested only once in a microwear study of otters, where it showed no interspecific differences (Beatty and Bao 2025). Our results provide the first evidence that Sdd may capture dietary variation, with higher values linked to harder food consumption. These previous studies allow us to link the differences seen between our samples to differences in material properties of diet, specifically harder diets among wolves from MIS 5e and Poland.

More complex dental textures in Polish and MIS 5e wolves could theoretically arise from eating small mammals, birds or invertebrates with hard parts. However, scat analyses show that such foods form only a minor, mainly summer, component of wolf diets (Ciucci et al. 1996; Nowak et al. 2005, 2011). We therefore interpret mild winters as the primary driver of the behavioural shift toward more intensive carcass utilisation. In contrast, the cooler climates of MIS 7a–c reduced reliance on bone, producing microwear signatures consistent with minimal hard‐tissue consumption.

Today, severe winters are known to be advantageous for wolves, since prey species are weakened by reduced access to ground vegetation and slower to escape over snow and ice, leading to enhanced hunting success (Gable et al. 2018; Jędrzejewski et al. 1992; Mech and Peterson 2003). Alternatively, during mild winters, ungulate prey tends to be in better physical condition, making it harder and energetically more expensive to hunt (Vucetich et al. 2012). This leads to wolves scavenging more intensively and consuming greater proportions of carcasses during milder winters, independent of kill rate and pack size (Gable et al. 2024). Dental microwear analyses suggest that decreasing winter severity under recent climatic warming has influenced wolf dietary behaviour, as indicated by comparisons of populations from Alaska and the Greater Yellowstone Ecosystem (GYE). Modern wolves in the GYE exhibited higher complexity values, consistent with increased carcass consumption (Burtt and DeSantis 2022), coinciding with a 5.4% reduction in Northern Hemisphere snow cover between 1972 and 2006 C.E. (Rasouli et al. 2015) and rising mean temperatures in the GYE since 1950 (Hansen and Phillips 2018). The same underlying climatic drivers are present in Poland, with a consistent and prolonged trend of increasing temperatures observed over the last 65 years and a mean annual increase of 0.25°C–0.40°C per decade (Kubiak‐Wójcicka and Machula 2020). More frequent rainfall instead of snowfall has been observed in Poland from the late 20th and early 21st centuries and the snow cover season has shortened considerably, particularly in the western and central regions (5–7 days/decade), with winter temperatures now above or close to 0°C (Wibig and Jędruszkiewicz 2023).

Continued climatic warming is anticipated to further reduce winter snow cover, with implications for both wolves and ecosystems on a global scale. Critically, longer ice‐free periods are expected to alter wolf‐prey dynamics, for example, leading to new intensification of predation on certain species such as beaver and reducing late winter carrion availability, with negative consequences for scavengers (Gable et al. 2018). A key question is therefore whether these patterns in wolf predation will continue into the future or whether they will change.

Our results suggest that wolves in Poland are processing carcasses in a way that is most comparable to that seen in wolves from past periods of elevated global temperatures. The last interglacial may therefore serve as an analogue for near‐future climate scenarios. In this respect, the grey wolf in Poland may benefit from its exceptional ability to exploit the human environment, given the current increase in the number and biomass of wild ungulate populations in agricultural areas (Valente et al. 2020) and the ample supply of carrion ensured by traffic collisions (Grilo et al. 2025), game hunting and diseases at the wildlife‐domestic interface (Abrantes and Vieira‐Pinto 2023). While this offers considerable hope for the future success of the grey wolf in Poland, the same conditions may not be applicable for other vulnerable populations, especially those more remote from humanly‐modified environments.

Evidence of changes in dietary behaviour, specifically increased durophagy, may therefore warn wildlife managers of climate‐related stress in warming regions. In conclusion, we would argue that the threat of climate warming, and particularly a reduction in winter severity, should be factored into future conservation planning for this species.

## Author Contributions

Conceptualization: D.C.S. and A.L.L.; Methodology: D.C.S., A.A.B. and M.A.P.; Data collection: A.A.B.; Poland specimen collection and processing: S.N., R.W.M. and M.F.; Data analysis: A.A.B., N.F.A. and M.A.P.; Writing – original draft: D.C.S., A.A.B., N.F.A., S.N. and R.W.M.; Writing – review and editing: D.C.S., A.A.B., N.F.A., S.N., R.W.M., M.A.P. and A.L.L.; All authors read and approved the final manuscript.

## Funding

This work was supported by the Ministry of Education and Science, Poland, No. DWD/5/0413/2021; Natural Environment Research Council, NE/W006103/1; National Science Centre, Poland, No. 2024/55/B/NZ9/0269.

## Supporting information


**Figure S1:** ele70337‐sup‐0001‐FigureS1.docx.

**Appendix S1:** ele70337‐sup‐0002‐AppendixS1.docx.

## Data Availability

All data supporting these results has been deposited in the NERC EDS National Geoscience Data Centre. (Dataset). https://doi.org/10.5285/0aa0e3e8‐d668‐4dd4‐9089‐7bd0ede798d7.
