# Supplementary material for: Climate Change Challenges Grey Wolf Resilience: Insights From Dental Microwear
Source: Ecol Lett. 2026 Feb 11;29(2):e70337. doi: 10.1111/ele.70337 (PMC12893404; doi:10.1111/ele.70337)
Supplement: Supplementary file 1 — Figure S1: ele70337‐sup‐0001‐FigureS1.docx. [file ELE-29-0-s002.docx]

Supporting Information – Figure legend


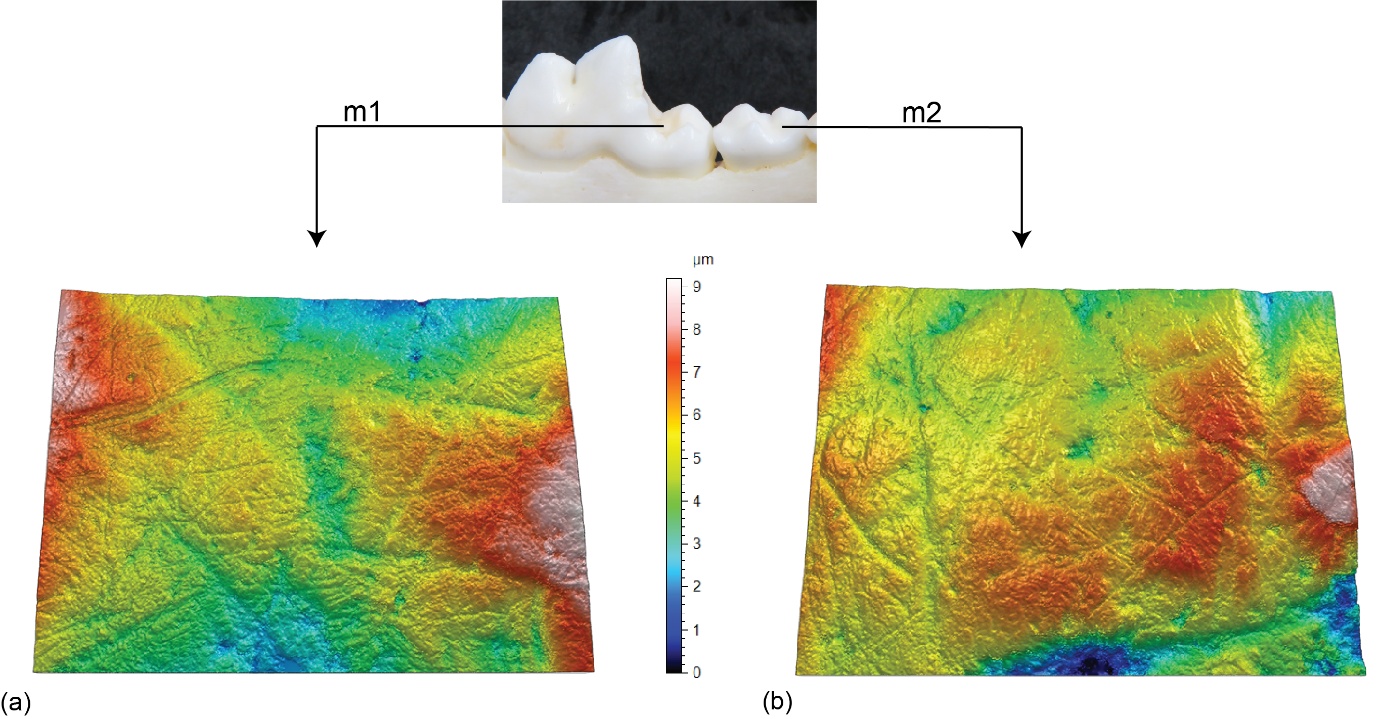


| Figure S1 | Rendered scans of wolf tooth surfaces with topographic contouring, illustrating m1 and m2 tooth surfaces from the same specimens (a) m1 scan from Torquay Museum specimen Joint Mitnor Cave TORMSP35085 (Asfc = 12.23; NewEplsar = 0.01795), (b) m2 scan from specimen Joint Mitnor Cave TORMSP35085 (Asfc = 10.41; NewEplsar = 0.01757). |
| --- | --- |
