# Supplementary material for: Climate Change Challenges Grey Wolf Resilience: Insights From Dental Microwear
Source: Ecol Lett. 2026 Feb 11;29(2):e70337. doi: 10.1111/ele.70337 (PMC12893404; doi:10.1111/ele.70337)
Supplement: Supplementary file 2 — Appendix S1: ele70337‐sup‐0002‐AppendixS1.docx. [file ELE-29-0-s001.docx]

Supporting Information

**Title**: Climate change challenges grey wolf resilience: insights from dental microwear

**Authors**: Amanda A. Burtt^1^, Neil F. Adams^2, 3^, Sabina Nowak^4^, Robert W. Mysłajek^4^ Michal Figura^4^, Mark A. Purnell^3^, Angela L. Lamb^5^, Danielle C. Schreve^1^

Affiliations:

^1^ School of Geographical Sciences, University of Bristol, University Road, Bristol, BS8 1SS, United Kingdom. Emails: [amanda.burtt@bristol.ac.uk](mailto:amanda.burtt@bristol.ac.uk), [danielle.schreve@bristol.ac.uk](mailto:danielle.schreve@bristol.ac.uk)

^2^ Natural History Museum, Cromwell Road, London, SW7 5BD. Email: [n.adams@nhm.ac.uk](mailto:n.adams@nhm.ac.uk)

^3^ Centre for Palaeobiology and Biosphere Evolution, School of Geography, Geology and the Environment, University of Leicester, University Road, Leicester, LE1 7RH. Email: [map2@leicester.ac.uk](mailto:map2@leicester.ac.uk)

^4^ University of Warsaw, Faculty of Biology, Institute of Ecology, Department of Animal Ecology and Evolution, Żwirki i Wigury 101, 02-089 Warszawa, Poland. E-mails: [sabina.pieruzeknowak@gmail.com](mailto:sabina.pieruzeknowak@gmail.com), [r.myslajek@uw.edu.pl](mailto:r.myslajek@uw.edu.pl), [figura.michal@gmail.com](mailto:figura.michal@gmail.com)

^5^ British Geological Survey, Nottingham, United Kingdom. Email: [alla@bgs.ac.uk](mailto:alla@bgs.ac.uk)

All data supporting these results has been deposited in the NERC EDS National Geoscience Data Centre. (Dataset). <https://doi.org/10.5285/0aa0e3e8-d668-4dd4-9089-7bd0ede798d7>

Sites and materials

Well-preserved dental material from the British Pleistocene wolves was selected from sites for which there is an established robust chronology (Supporting Information, Table S1), based on mammalian biostratigraphy (Currant & Jacobi 2011; Schreve 2001) and supplemented, where available, by radiometric dating. We follow the methodology of Berger et al. (2015) in terms of establishing criteria for the diagnosis of interglacials in the last 800 000 years. This identifies MIS 7a-c as a distinct, single interglacial period, separate to MIS 7e.

For MIS 5e, the sites were Durdham Down in Bristol, housed at Bristol Museum and Art Gallery and the Natural History Museum, London (NHMUK), and Joint Mitnor Cave in Devon, housed at Torquay Museum and the NHMUK. Both sites contain a mammalian assemblage consistent with the Joint Mitnor Cave Mammal Assemblage-Zone (MAZ) of Currant and Jacobi (2011). Durdham Down consisted of a now-lost fissure deposit, exposed during quarrying of Carboniferous limestone in the early part of the 19^th^ century. Little is known of the stratigraphy, but the sediments yielded remains of a Last Interglacial temperate-climate faunal assemblage, including typical species such as hippopotamus (*Hippopotamus amphibius*), narrow-nosed rhinoceros (*Stephanorhinus hemitoechus*) and straight-tusked elephant (*Palaeoloxodon antiquus*), interpreted as the remains of a spotted hyaena (*Crocuta crocuta*) den (Wilson 1885; Donovan 1954 and references therein). Joint Mitnor Cave, located within an outcrop of Devonian limestone at around 65m O.D., was discovered in 1939 and consisted of up to 1.5m of fossil-rich “cave earth” and breccia, overlying sterile waterlain sediments, and capped by a stalagmite floor (Sutcliffe 1958). The rich faunal assemblage was recovered from a talus cone, forming below a fissure in the cave roof, allowing for hippopotamus, straight-tusked elephant, fallow deer (*Dama dama*) and other species to enter through a natural pitfall trap (Sutcliffe 1958).

For MIS 7a-c, the sites were Bleadon Cavern housed at the Somerset Heritage Centre (South West Heritage Trust), Hutton Cave, housed at the Somerset Heritage Centre (South West Heritage Trust) and the NHMUK, Pontnewydd Cave, Clwyd, housed at the National Museum Wales in Cardiff, together with Ilford in Essex, housed at the British Geological Survey (Davies 1874) and Marsworth, Buckinghamshire, housed at Bucks Museum Resource Centre (Murton et al. 2001).

Mammalian assemblages from MIS 7a-c in Britain are characterised by a predominance of open-ground indicators such as horse (*Equus ferus*), a late morphotype of steppe mammoth (*Mammuthus trogontherii*) and narrow-nosed rhinoceros, designated as the Sandy Lane MAZ (Schreve 2001). Bleadon Cavern, excavated from 1833 onwards (Currant 2004), is located on the northern edge of the Mendip Hills in Somerset, in Carboniferous limestone and lying at c.100m O.D. Extremely rich collections of bones were recovered from a debris flow and include large numbers of *Panthera spelaea* (cave lion), horse, straight-tusked elephant and steppe mammoth (Schreve 1998). Hutton Cave, also known as Upper Canada Cave and located on the same hillside as Bleadon, was discovered by ochre miners c.1756 (Catcott 1761). It contains a very similar fauna, also attributed to the Sandy Lane MAZ by Schreve (2001), and including large numbers of wolf remains. Uranium-series dating of a flowstone apparently emplaced above the bone breccia in Bleadon Cave produced an age of 230±7 ka BP (Hodge et al. 2016). This would suggest that the bone assemblage was laid down during either the stadial conditions of MIS 7d or the MIS 7e interglacial, but the faunal composition is a poor match for MIS 7e and the assemblage contains many temperate-climate species that equally do not correspond with cold climate stadial conditions (Schreve 1998, 2001). An age within MIS 7a-c is therefore preferred (Currant 2004). Pontnewydd Cave, located within an outcrop of Carboniferous limestone at 89.5m O.D., was first discovered in 1832 but excavated extensively in the latter half of the 20^th^ century (Green 1984, 1995). The bone assemblage, which includes remains of Neanderthals (*Homo neanderthalensis*), comes predominantly from a debris flow, the “Lower Breccia” and includes narrow-nosed rhinoceros, Merck’s rhinoceros (*Stephanorhinus kirchbergensis*) and horse (Schreve 1998). Thermoluminescence dating of burnt flints from within the Lower Breccia and Uranium-series dating of stalagmitic floors on top of this deposit suggest an age of around 200ka B.P. (Huxtable 1984; Schwarcz 1984).

The final two sites from MIS 7a-c are open (fluvial) sites. Faunal remains from Ilford in Essex came to light during quarrying for “brickearth” (silty clay) during the early 19^th^ century (Davies 1874). The fossiliferous deposits are centred on 7m O.D. and lie within the Mucking Gravel Formation of the Lower Thames terrace staircase sequence (Bridgland 1994). Interglacial deposits within that terrace have been correlated with MIS 7, using lithostratigraphy (Bridgland 1994), molluscan biostratigraphy (Keen 2001) and aminostratigraphy (Penkman et al. 2013), as well as mammalian biostratigraphy, based on the combined presence of horse, steppe mammoth, narrow-nosed rhinoceros and Merck’s rhinoceros (Schreve 2001). The deposits of the Lower Channel at Marsworth consist of gravelly, calcareous sands, organic muds and tufaceous deposits, laid down by a spring-fed stream at the foot of the Chiltern Hills, c.124-126m OD. The stratigraphic position of the Lower Channel (Murton et al. 2001), together with evidence from mammalian biostratigraphy (Schreve 2001), aminostratigraphy (Penkman et al. 2013) and absolute geochronology (Murton et al. 2001; Candy and Schreve 2007) indicate an age within MIS 7a-c.

Poland wolves

The sample from Poland, housed in the University of Warsaw, comprised 24 individual wolves (13 females and 11 males), collected between 2001 and 2023 from six different regions (Supporting Information, Table S2). These include the Carpathian Mountains (*n* = 8), West Poland (*n* = 7), Northwest Poland (*n* = 3), Central Poland (*n* = 1), Northeast Poland (*n* = 3), and East Poland (*n* = 2). No significant differences in SSFA parameters or ISO parameters were observed among regions nor detected between males and females. Wolves from Poland were assigned to age classes based on tooth wear (Gipson et al. 2000). We divided wolves into 2 groups, wolves under and including the age class 3–4 (*n* = 15) and those over and including the 4–6 age class (*n* = 9) and no significant differences were detected for SSFA parameters (*Asfc*: *Z* = 0.060, *p* = 0.953; *NewEpLsar*: *Z* = 0.119, *p* = 0.905)*.*We chose to separate wolves based on being above or below four years of age because once advanced beyond approximately four years of age visible wear is visible on the tip of all carnassial prominences (Gipson et al. 2000). No differences were detected between wolf specimens collected in different seasons. To test for season effects on diets, we divided date of death for Poland wolves into October–March and April–September aligning with common seasonal differences (Peel et al. 2007) (Supporting Information, Tables S18-S19).

Statistics

Benjamini-Hochberg procedure was employed for each statistical analysis to take into account the possibility that some results are significant because of the increased type I error associated with multiple comparisons (Benjamini and Hochberg 1995). The false discovery rate was set at 0.05. This is a particularly powerful approach and is preferable to approaches that seek to control the family-wise error rate, such as the Bonferroni correction. The only exception to this was pairwise testing using the Steel-Dwass pairwise test, since this test corrects for the inflated type I error rates associated with multiple comparisons.

All statistical analyses were carried out in JMP Pro 18.2.1 Student Edition (JMP Statistical Discovery LLC, Cary, NC USA), except for the Benjamini-Hochberg procedure, which used Microsoft Excel (McDonald 2014; <http://www.biostathandbook.com/benjaminihochberg.xls>).

Supporting Information Table Legend

| Referenced in Main test | |
| --- | --- |
| Table S1 | Descriptive data for British fossil wolves. |
| Table S2 | Poland wolf ID, region location of carcass, sex, and age class. |
| Table S3 | Matched-pair comparisons of first and second molar SSFA texture parameters in Poland wolves; significance was assessed using the Benjamini–Hochberg procedure (FDR = 0.05). |
| Table S4 | Matched-pair comparisons of first and second molar SSFA texture parameters in Poland and Britain wolves; significance was assessed using the Benjamini–Hochberg procedure (FDR = 0.05). |
| Table S5 | Matched-pair comparisons of first and second molar areal texture parameters in Poland wolves; significance was assessed using the Benjamini–Hochberg procedure (FDR = 0.05). |
| Table S6 | Matched-pair comparisons of first and second molar areal texture parameters in Poland and Britain wolves; significance was assessed using the Benjamini–Hochberg procedure (FDR = 0.05). |
| Table S7 | Wilcoxon two-sample tests comparing SSFA texture parameters between MIS 5e and MIS 7a–c wolves; significance was assessed using the Benjamini–Hochberg procedure (FDR = 0.05). |
| Table S8 | Wilcoxon two-sample tests comparing areal texture parameters between MIS 5e and MIS 7a–c wolves; significance was assessed using the Benjamini–Hochberg procedure (FDR = 0.05). |
| Table S9. | Eigenvalues from PCA of 19 areal texture parameters that differ significantly between MIS 5e and MIS 7a–c following application of the Benjamini–Hochberg procedure. |
| Table S10 | Eigenvectors from PCA of 19 areal texture parameters that differ between MIS 5e and MIS 7a–c. |
| Table S11 | Kruskal–Wallis tests comparing SSFA texture parameters among MIS 5e, MIS 7a–c, and modern Polish wolves; significance was assessed using the Benjamini–Hochberg procedure (FDR = 0.05). |
| Table S12 | Descriptive statistics of SSFA complexity and anisotropy values from all modern and Pleistocene wolves examined. |
| Table S13 | Results comparing complexity and anisotropy parameters among MIS 5e, MIS 7a–c, and modern Polish wolves based on Kruskal–Wallis tests and subsequent Steel–Dwass pairwise comparisons. |
| Table S14 | Kruskal–Wallis tests comparing areal texture parameters among MIS 5e, MIS 7a–c, and modern Polish wolves; significance was assessed using the Benjamini–Hochberg procedure (FDR = 0.05). |
| Table S15 | Eigenvalues from PCA of 30 areal texture parameters that differ significantly between MIS 5e, MIS 7a–c, and modern Polish wolves following application of the Benjamini–Hochberg procedure (FDR = 0.05).. |
| Table S16 | Eigenvectors from PCA of 30 areal texture parameters that differ between MIS 5e, MIS 7a–c, and modern Polish wolves. |
| Table S17 | PCA of 30 areal texture parameters that differ between MIS 5e, MIS 7a–c, and modern Polish wolves; Steel–Dwass pairwise comparisons of PC 1 values. |
| Referenced in Supporting Information only | |
| Table S18 | Kruskal–Wallis tests* comparing SSFA texture parameters among Polish regions, and Wilcoxon two-sample tests comparing SSFA texture parameters by season (October–March vs. April–September), age, and sex. No parameters remained significant following application of the Benjamini–Hochberg procedure (FDR = 0.05). |
| Table S19 | Kruskal–Wallis tests* comparing areal texture parameters among Polish regions, and Wilcoxon two-sample tests comparing ISO texture parameters by season (October–March vs. April–September), age, and sex. No parameters remained significant following application of the Benjamini–Hochberg procedure (FDR = 0.05). |

Supporting Information Tables

Table S1. Descriptive data for British fossil wolves.

| Store location | Sites | tooth | MIS | Specimen number |
| --- | --- | --- | --- | --- |
| MIS 7(a-c) |  |  |  |  |
| Bucks Museum Resource Centre | Marsworth | m1 | 7 | AYBCM 1980.544.G9/12A |
| Bucks Museum Resource Centre | Marsworth | m1 | 7 | AYBCM 1980.544.H8/9 |
| Bucks Museum Resource Centre | Marsworth | m1 | 7 | AYBCM 1980.544.H9/3 |
| National Museum, Wales | Pontnewydd Cave | m1 | 7 | 86.31H/3.D3990 |
| National Museum, Wales | Pontnewydd Cave | m1 | 7 | 88.166H/3.H498.1 |
| Somerset Archaeological and Natural History Society and  South West Heritage Trust | Bleadon Cavern | m1 | 7 | TTNCM: 41/1995/12 |
| UKBGS | Ilford | m2 | 7 | GSM 95739 (204) |
| Bucks Museum Resource Centre | Marsworth | m2 | 7 | AYBCM 1980.544.L6/2 |
| National Museum, Wales | Pontnewydd Cave | m2 | 7 | 84.70H/2.D3765 |
| National Museum, Wales | Pontnewydd Cave | m2 | 7 | 95.35H/3.F5291 |
| National Museum, Wales | Pontnewydd Cave | m2 | 7 | 86.31H/3.D4144 |
| Somerset Archaeological and Natural History Society and  South West Heritage Trust | Hutton Cave | m2 | 7 | TTNCM: 42/1995/44 |
| NHMUK | Hutton Cave | m2 | 7 | M108980b |
| MIS 5e |  |  |  |  |
| Bristol Museum | Durdham Down Cave | m1 | 5e | Cg2664 |
| Bristol Museum | Durdham Down Cave | m1 | 5e | Cg2666 |
| Bristol Museum | Durdham Down Cave | m1 | 5e | Cg2667 |
| NHMUK | Durdham Down Cave | m1 | 5e | NHMUK PV M 108993 |
| TORMS | Joint Mitnor | m1 | 5e | P35113 |
| TORMS | Joint Mitnor | m1 | 5e | P35114 |
| Bristol Museum | Durdham Down Cave | m2 | 5e | Cg3020 |
| TORMS | Joint Mitnor | m2 | 5e | P35082 |
| TORMS | Joint Mitnor | m2 | 5e | P35085 |
| TORMS | Joint Mitnor | m2 | 5e | P35050 |
| TORMS | Joint Mitnor | m2 | 5e | P35048 |
| NHMUK | Durdham Down Cave | m2 | 5e | NHMUK PV M 108991 |
| NHMUK | Joint Mitnor | m2 | 5e | NHMUK PV M 109012 |

Table S2. Poland wolf ID, region location of carcass, sex, and age class.

| Wolf ID | Poland region | Sex | Age class |
| --- | --- | --- | --- |
| F.2017.10.03 | Carpathian Mts | Female | 8-10 |
| F.2001.04.23 | Carpathian Mts | Female | 2-3 |
| F.2015.08.23 | Carpathian Mts | Female | 4-6 |
| F.2016.01.15 | Carpathian Mts | Female | 6-8 |
| M.2021.04.05 | Carpathian Mts | Male | 2-3 |
| M.2023.03.15 | Carpathian Mts | Male | 1-2 |
| M.2020.07.22 | Carpathian Mts | Male | 3-4 |
| M.2019.09.22 | Carpathian Mts | Male | 2-3 |
| M.2016.04.17 | Central Poland | Male | 1-2 |
| F.2021.10.06 | East Poland | Female | 4-6 |
| F.2022.02.24 | East Poland | Female | 8-10 |
| F.2019.03.21 | Northeast Poland | Female | 4-6 |
| F.2018.04.17 | Northeast Poland | Female | 3-4 |
| M.2017.02.14 | Northeast Poland | Male | 2-3 |
| M.2019.02.19 | Northwest Poland | Male | 3-4 |
| M.2017.06.21 | Northwest Poland | Male | 2-3 |
| M.2023.02.01 | Northwest Poland | Male | 2-3 |
| F.2022.11.04 | West Poland | Female | 2-3 |
| F.2018.10.18 | West Poland | Female | 4-6 |
| F.2017.03.21 | West Poland | Female | 6-8 |
| F.2017.01.30 | West Poland | Female | 6-8 |
| F.2019.10.21 | West Poland | Female | 1-2 |
| M.2015.06.16 | West Poland | Male | 3-4 |
| M.2022.02.22 | West Poland | Male | 1-2 |

Table S3. Matched-pair comparisons of first and second molar SSFA texture parameters in Poland wolves; significance was assessed using the Benjamini–Hochberg procedure (FDR = 0.05).

| SSFA parameter | *S* | *p* | Benjamini-Hochberg significance |
| --- | --- | --- | --- |
| Area-scale (one corner) |  |  |  |
| Fractal complexity (Asfc) | 2.5 | 0.820 | not significant |
| Fractal dimension (Das) | 2.5 | 0.820 | not significant |
| HAsfc9 (HAsfc9) | 1.5 | 0.910 | not significant |
| HAsfc81 (HAsfc81) | 0.5 | 1.000 | not significant |
| Heterogeneity of Asfc (HAsfc) | 1.5 | 0.910 | not significant |
| Mean of Asfc (MeanAsfc) | 0.5 | 1.000 | not significant |
| Median absolute deviation of Asfc (MadAsfc) | 1.5 | 0.910 | not significant |
| Median of Asfc (MedianAsfc) | 1.5 | 0.910 | not significant |
| Smooth-rough crossover (SRC) | 13.5 | 0.129 | not significant |
| Standard deviation of Asfc (StdDevAsfc) | 1.5 | 0.910 | not significant |
| Length-scale (rows) |  |  |  |
| Fractal complexity (Lsfc) | 0.5 | 1.000 | not significant |
| Fractal dimension (Dls) | 0.5 | 1.000 | not significant |
| Length-scale anisotropy (NewEplsar) | 9.5 | 0.301 | not significant |
| Length-scale anisotropy (Sfrax) (epLsar) | 7.5 | 0.426 | not significant |
| Scale of max complexity (Smfc) | 15.5 | 0.074 | not significant |
| Smooth-rough crossover (SRC) | 18.5 | 0.027 | not significant |

Table S4. Matched-pair comparisons of first and second molar SSFA texture parameters in Poland and Britain wolves; significance was assessed using the Benjamini–Hochberg procedure (FDR = 0.05).

| SSFA parameter | *S* | *p* | Benjamini-Hochberg significance |
| --- | --- | --- | --- |
| Area-scale (one corner) |  |  |  |
| Fractal complexity (Asfc) | 19 | 0.151 | not significant |
| Fractal dimension (Das) | 19 | 0.151 | not significant |
| HAsfc9 (HAsfc9) | 5 | 0.733 | not significant |
| HAsfc81 (HAsfc81) | 12 | 0.380 | not significant |
| Heterogeneity of Asfc (HAsfc) | 5 | 0.733 | not significant |
| Mean of Asfc (MeanAsfc) | 12 | 0.380 | not significant |
| Median absolute deviation of Asfc (MadAsfc) | 12 | 0.380 | not significant |
| Median of Asfc (MedianAsfc) | 15 | 0.266 | not significant |
| Scale of max complexity (Smfc) | 0 | 1.000 | not significant |
| Smooth-rough crossover (SRC) | 11 | 0.424 | not significant |
| Standard deviation of Asfc (StdDevAsfc) | 6 | 0.677 | not significant |
| Length-scale (rows) |  |  |  |
| Fractal complexity (Lsfc) | 8 | 0.569 | not significant |
| Fractal dimension (Dls) | 8 | 0.569 | not significant |
| Length-scale anisotropy (NewEplsar) | 2 | 0.910 | not significant |
| Length-scale anisotropy (Sfrax) (epLsar) | 20 | 0.129 | not significant |
| Scale of max complexity (Smfc) | 9 | 0.519 | not significant |
| Smooth-rough crossover (SRC) | 16 | 0.233 | not significant |

Table S5. Matched-pair comparisons of first and second molar areal texture parameters in Poland wolves; significance was assessed using the Benjamini–Hochberg procedure (FDR = 0.05).

| ISO parameter | *S* | *p* | Benjamini-Hochberg significance |
| --- | --- | --- | --- |
| S5p | 6.5 | 0.496 | not significant |
| S5v | 14.5 | 0.098 | not significant |
| S10z | 6.5 | 0.496 | not significant |
| Sa | 18.5 | 0.027 | not significant |
| Sak1 | 9.5 | 0.301 | not significant |
| Sak2 | 6.5 | 0.496 | not significant |
| Sal | 9.5 | 0.301 | not significant |
| Sda | 4.5 | 0.652 | not significant |
| Sdaq | 2.5 | 0.820 | not significant |
| Sdar | 10.5 | 0.250 | not significant |
| Sdarq | 10.5 | 0.250 | not significant |
| Sdarx | 14.5 | 0.098 | not significant |
| Sdax | 7.5 | 0.426 | not significant |
| Sdc | 17.5 | 0.039 | not significant |
| Sdd | 5.5 | 0.570 | not significant |
| Sddq | 2.5 | 0.820 | not significant |
| Sddx | 9.5 | 0.301 | not significant |
| Sded | 7.5 | 0.426 | not significant |
| Sdedq | 0.5 | 1.000 | not significant |
| Sdedx | 7.5 | 0.426 | not significant |
| Sdff | 15.5 | 0.074 | not significant |
| Sdffq | 8.5 | 0.359 | not significant |
| Sdffx | 7.5 | 0.426 | not significant |
| Sdn | 2.5 | 0.820 | not significant |
| Sdq | 8.5 | 0.359 | not significant |
| Sdr | 11.5 | 0.203 | not significant |
| Sdrn | 6.5 | 0.496 | not significant |
| Sdrnq | 8.5 | 0.359 | not significant |
| Sdrnx | 9.5 | 0.301 | not significant |
| Sdv | 0.5 | 1.000 | not significant |
| Sdvq | 1.5 | 0.910 | not significant |
| Sdvx | 1.5 | 0.910 | not significant |
| Sha | 7.5 | 0.426 | not significant |
| Shaq | 7.5 | 0.426 | not significant |
| Shar | 0.5 | 1.000 | not significant |
| Sharq | 0.5 | 1.000 | not significant |
| Sharx | 0.5 | 1.000 | not significant |
| Shax | 0.5 | 1.000 | not significant |
| Shed | 3.5 | 0.734 | not significant |
| Shedq | 8.5 | 0.359 | not significant |
| Shedx | 0.5 | 1.000 | not significant |
| Shff | 7.5 | 0.426 | not significant |
| Shffq | 7.5 | 0.426 | not significant |
| Shffx | 1.5 | 0.910 | not significant |
| Shh | 4.5 | 0.652 | not significant |
| Shhq | 0.5 | 1.000 | not significant |
| Shhx | 1.5 | 0.910 | not significant |
| Shn | 9.5 | 0.301 | not significant |
| Shrn | 1.5 | 0.910 | not significant |
| Shrnq | 0.5 | 1.000 | not significant |
| Shrnx | 3.5 | 0.734 | not significant |
| Shv | 5.5 | 0.570 | not significant |
| Shvq | 5.5 | 0.570 | not significant |
| Shvx | 9.5 | 0.301 | not significant |
| Sk | 13.5 | 0.129 | not significant |
| Sku | 5.5 | 0.570 | not significant |
| Smc | 17.5 | 0.039 | not significant |
| Smq | 10.5 | 0.250 | not significant |
| Smr | 6.5 | 0.496 | not significant |
| Smrk1 | 4.5 | 0.652 | not significant |
| Smrk2 | 0.5 | 1.000 | not significant |
| Sp | 6.5 | 0.496 | not significant |
| Spc | 1.5 | 0.910 | not significant |
| Spd | 9.5 | 0.301 | not significant |
| Spk | 9.5 | 0.301 | not significant |
| Spkx | 9.5 | 0.301 | not significant |
| Spq | 15.5 | 0.074 | not significant |
| Sq | 15.5 | 0.074 | not significant |
| Ssk | 8.5 | 0.359 | not significant |
| Ssw | 14.5 | 0.098 | not significant |
| Std | 5.5 | 0.570 | not significant |
| Str | 4.5 | 0.652 | not significant |
| Sv | 14.5 | 0.098 | not significant |
| Svc | 0.5 | 1.000 | not significant |
| Svd | 4.5 | 0.652 | not significant |
| Svk | 10.5 | 0.250 | not significant |
| Svkx | 14.5 | 0.098 | not significant |
| Svq | 9.5 | 0.301 | not significant |
| Sz | 3.5 | 0.734 | not significant |
| Vm | 9.5 | 0.301 | not significant |
| Vmc | 15.5 | 0.074 | not significant |
| Vmp | 9.5 | 0.301 | not significant |
| Vv | 16.5 | 0.055 | not significant |
| Vvc | 12.5 | 0.164 | not significant |
| Vvv | 11.5 | 0.203 | not significant |

Table S6. Matched-pair comparisons of first and second molar areal texture parameters in Poland and Britain wolves; significance was assessed using the Benjamini–Hochberg procedure (FDR = 0.05).

| ISO parameter | *S* | *p* | Benjamini-Hochberg significance |
| --- | --- | --- | --- |
| S5p | 13.0 | 0.339 | not significant |
| S5v | 4.0 | 0.791 | not significant |
| S10z | 2.0 | 0.910 | not significant |
| Sa | 16.0 | 0.233 | not significant |
| Sak1 | 10.0 | 0.470 | not significant |
| Sak2 | 2.0 | 0.910 | not significant |
| Sal | 7.0 | 0.622 | not significant |
| Sda | 14.0 | 0.301 | not significant |
| Sdaq | 6.0 | 0.677 | not significant |
| Sdar | 24.0 | 0.064 | not significant |
| Sdarq | 26.0 | 0.042 | not significant |
| Sdarx | 30.0 | 0.016 | not significant |
| Sdax | 7.0 | 0.622 | not significant |
| Sdc | 26.0 | 0.042 | not significant |
| Sdd | 8.0 | 0.569 | not significant |
| Sddq | 7.0 | 0.622 | not significant |
| Sddx | 5.0 | 0.733 | not significant |
| Sded | 16.0 | 0.233 | not significant |
| Sdedq | 12.0 | 0.380 | not significant |
| Sdedx | 7.0 | 0.622 | not significant |
| Sdff | 20.0 | 0.129 | not significant |
| Sdffq | 4.0 | 0.791 | not significant |
| Sdffx | 3.0 | 0.850 | not significant |
| Sdn | 11.0 | 0.424 | not significant |
| Sdq | 2.0 | 0.910 | not significant |
| Sdr | 14.0 | 0.301 | not significant |
| Sdrn | 13.0 | 0.339 | not significant |
| Sdrnq | 15.0 | 0.266 | not significant |
| Sdrnx | 4.0 | 0.791 | not significant |
| Sdv | 6.0 | 0.677 | not significant |
| Sdvq | 2.0 | 0.910 | not significant |
| Sdvx | 7.0 | 0.622 | not significant |
| Sha | 11.0 | 0.424 | not significant |
| Shaq | 23.0 | 0.077 | not significant |
| Shar | 9.0 | 0.519 | not significant |
| Sharq | 1.0 | 0.970 | not significant |
| Sharx | 3.0 | 0.850 | not significant |
| Shax | 17.0 | 0.204 | not significant |
| Shed | 0.0 | 1.000 | not significant |
| Shedq | 22.0 | 0.092 | not significant |
| Shedx | 17.0 | 0.204 | not significant |
| Shff | 2.0 | 0.910 | not significant |
| Shffq | 17.0 | 0.204 | not significant |
| Shffx | 2.0 | 0.910 | not significant |
| Shh | 4.0 | 0.791 | not significant |
| Shhq | 8.0 | 0.569 | not significant |
| Shhx | 6.0 | 0.677 | not significant |
| Shn | 17.0 | 0.204 | not significant |
| Shrn | 13.0 | 0.339 | not significant |
| Shrnq | 9.0 | 0.519 | not significant |
| Shrnx | 2.0 | 0.910 | not significant |
| Shv | 11.0 | 0.424 | not significant |
| Shvq | 8.0 | 0.569 | not significant |
| Shvx | 1.0 | 0.970 | not significant |
| Sk | 24.0 | 0.064 | not significant |
| Sku | 7.0 | 0.622 | not significant |
| Smc | 20.0 | 0.129 | not significant |
| Smq | 8.0 | 0.569 | not significant |
| Smr | 16.0 | 0.233 | not significant |
| Smrk1 | 5.0 | 0.733 | not significant |
| Smrk2 | 8.0 | 0.569 | not significant |
| Sp | 19.0 | 0.151 | not significant |
| Spc | 16.0 | 0.233 | not significant |
| Spd | 17.0 | 0.204 | not significant |
| Spk | 11.0 | 0.424 | not significant |
| Spkx | 22.0 | 0.092 | not significant |
| Spq | 21.0 | 0.110 | not significant |
| Sq | 11.0 | 0.424 | not significant |
| Ssk | 5.0 | 0.733 | not significant |
| Ssw | 25.0 | 0.052 | not significant |
| Std | 16.0 | 0.233 | not significant |
| Str | 15.0 | 0.266 | not significant |
| Sv | 4.0 | 0.791 | not significant |
| Svc | 10.0 | 0.470 | not significant |
| Svd | 13.0 | 0.339 | not significant |
| Svk | 2.0 | 0.910 | not significant |
| Svkx | 4.0 | 0.791 | not significant |
| Svq | 1.0 | 0.970 | not significant |
| Sz | 8.0 | 0.569 | not significant |
| Vm | 11.0 | 0.424 | not significant |
| Vmc | 26.0 | 0.042 | not significant |
| Vmp | 11.0 | 0.424 | not significant |
| Vv | 19.0 | 0.151 | not significant |
| Vvc | 23.0 | 0.077 | not significant |
| Vvv | 3.0 | 0.850 | not significant |

Table S7. Wilcoxon two-sample tests comparing SSFA texture parameters between MIS 5e and MIS 7a–c wolves; significance was assessed using the Benjamini–Hochberg procedure (FDR = 0.05).

| SSFA parameter | Z | p | Benjamini-Hochberg significance |
| --- | --- | --- | --- |
| Area-scale (one corner) |  |  |  |
| Smooth-rough crossover (SRC) | 0.000 | 1.000 | not significant |
| Fractal complexity (Asfc) | 4.205 | <0.0001 | significant |
| Fractal dimension (Das) | 4.205 | <0.0001 | significant |
| Scale of max complexity (Smfc) | 1.001 | 0.317 | not significant |
| HAsfc9 (HAsfc9) | 0.051 | 0.959 | not significant |
| HAsfc81 (HAsfc81) | 0.051 | 0.959 | not significant |
| Heterogeneity of Asfc (HAsfc) | 0.051 | 0.959 | not significant |
| Median of Asfc (MedianAsfc) | 4.154 | <0.0001 | significant |
| Mean of Asfc (MeanAsfc) | 4.308 | <0.0001 | significant |
| Standard deviation of Asfc (StdDevAsfc) | 3.231 | 0.001 | significant |
| Median absolute deviation of Asfc (MadAsfc) | 2.872 | 0.004 | significant |
| Length-scale (rows) |  |  |  |
| Smooth-rough crossover (SRC) | 0.718 | 0.473 | not significant |
| Fractal complexity (Lsfc) | 3.795 | <0.0001 | significant |
| Fractal dimension (Dls) | 3.795 | <0.0001 | significant |
| Scale of max complexity (Smfc) | 0.692 | 0.489 | not significant |
| Length-scale anisotropy (Sfrax) (epLsar) | 1.026 | 0.305 | not significant |
| Length-scale anisotropy (NewEplsar) | 0.872 | 0.383 | not significant |

Table S8. Wilcoxon two-sample tests comparing areal texture parameters between MIS 5e and MIS 7a–c wolves; significance was assessed using the Benjamini–Hochberg procedure (FDR = 0.05).

| ISO parameter | *Z* | *p* | Benjamini-Hochberg significance |
| --- | --- | --- | --- |
| Sq | 2.667 | 0.008 | significant |
| Ssk | 2.615 | 0.009 | significant |
| Sku | 2.103 | 0.036 | not significant |
| Sp | 0.872 | 0.383 | not significant |
| Sv | 2.923 | 0.003 | significant |
| Sz | 2.256 | 0.024 | not significant |
| Sa | 2.718 | 0.007 | significant |
| Smr | 0.718 | 0.473 | not significant |
| Smc | 2.821 | 0.005 | significant |
| Sdc | 2.308 | 0.021 | not significant |
| Sal | 0.821 | 0.412 | not significant |
| Str | 0.000 | 1.000 | not significant |
| Std | 2.205 | 0.027 | not significant |
| Ssw | 1.282 | 0.200 | not significant |
| Sdq | 4.256 | <0.0001 | significant |
| Sdr | 4.308 | <0.0001 | significant |
| Vm | 1.487 | 0.137 | not significant |
| Vv | 2.718 | 0.007 | significant |
| Vmp | 1.487 | 0.137 | not significant |
| Vmc | 2.051 | 0.040 | not significant |
| Vvc | 2.051 | 0.040 | not significant |
| Vvv | 2.923 | 0.003 | significant |
| Spd | 0.103 | 0.918 | not significant |
| Spc | 2.718 | 0.007 | significant |
| S10z | 3.487 | <0.001 | significant |
| S5p | 2.256 | 0.024 | not significant |
| S5v | 3.282 | 0.001 | significant |
| Sda | 0.718 | 0.473 | not significant |
| Sha | 0.154 | 0.878 | not significant |
| Sdv | 1.282 | 0.200 | not significant |
| Shv | 1.026 | 0.305 | not significant |
| Svd | 0.513 | 0.608 | not significant |
| Svc | 3.128 | 0.002 | significant |
| Shh | 2.564 | 0.010 | significant |
| Shhx | 0.923 | 0.356 | not significant |
| Shhq | 2.103 | 0.036 | not significant |
| Shax | 0.410 | 0.682 | not significant |
| Shaq | 0.564 | 0.573 | not significant |
| Shvx | 1.026 | 0.305 | not significant |
| Shvq | 1.179 | 0.238 | not significant |
| Sdd | 2.513 | 0.012 | not significant |
| Sddx | 2.821 | 0.005 | significant |
| Sddq | 3.179 | 0.001 | significant |
| Sdax | 0.205 | 0.837 | not significant |
| Sdaq | 0.615 | 0.538 | not significant |
| Sdvx | 0.051 | 0.959 | not significant |
| Sdvq | 0.821 | 0.412 | not significant |
| Shn | 0.000 | 1.000 | not significant |
| Sdn | 0.539 | 0.590 | not significant |
| Sk | 2.051 | 0.040 | not significant |
| Spk | 1.282 | 0.200 | not significant |
| Svk | 2.872 | 0.004 | significant |
| Smrk1 | 0.462 | 0.644 | not significant |
| Smrk2 | 1.231 | 0.218 | not significant |
| Spq | 1.795 | 0.073 | not significant |
| Svq | 2.103 | 0.036 | not significant |
| Smq | 0.000 | 1.000 | not significant |
| Sak1 | 1.282 | 0.200 | not significant |
| Sak2 | 2.872 | 0.004 | significant |
| Spkx | 0.718 | 0.473 | not significant |
| Svkx | 2.872 | 0.004 | significant |
| Shrn | 1.385 | 0.166 | not significant |
| Shrnx | 0.564 | 0.573 | not significant |
| Shrnq | 0.000 | 1.000 | not significant |
| Shff | 1.282 | 0.200 | not significant |
| Shffx | 0.769 | 0.442 | not significant |
| Shffq | 1.385 | 0.166 | not significant |
| Shed | 0.410 | 0.682 | not significant |
| Shedx | 0.410 | 0.682 | not significant |
| Shedq | 0.410 | 0.682 | not significant |
| Shar | 0.821 | 0.412 | not significant |
| Sharx | 0.564 | 0.573 | not significant |
| Sharq | 0.513 | 0.608 | not significant |
| Sdrn | 0.564 | 0.573 | not significant |
| Sdrnx | 1.231 | 0.218 | not significant |
| Sdrnq | 1.590 | 0.112 | not significant |
| Sdff | 0.308 | 0.758 | not significant |
| Sdffx | 2.000 | 0.046 | not significant |
| Sdffq | 1.744 | 0.081 | not significant |
| Sded | 0.564 | 0.573 | not significant |
| Sdedx | 0.205 | 0.837 | not significant |
| Sdedq | 0.821 | 0.412 | not significant |
| Sdar | 0.410 | 0.682 | not significant |
| Sdarx | 0.846 | 0.397 | not significant |
| Sdarq | 0.615 | 0.538 | not significant |

Table S9. Eigenvalues from PCA of 19 areal texture parameters that differ significantly between MIS 5e and MIS 7a–c following application of the Benjamini–Hochberg procedure.

| PC axis | Eigenvalue | Percent variance explained (%) | Cumulative percent (%) | *Z* | *p* | Benjamini-Hochberg significance |
| --- | --- | --- | --- | --- | --- | --- |
| PC 1 | 14.6595 | 77.1551 | 77.1551 | 3.282 | 0.001 | significant |
| PC 2 | 1.2882 | 6.7801 | 83.9352 | 0.769 | 0.442 | not significant |
| PC 3 | 1.0190 | 5.3632 | 89.2984 | 1.795 | 0.073 | not significant |
| PC 4 | 0.6526 | 3.4349 | 92.7333 | 0.667 | 0.505 | not significant |
| PC 5 | 0.4385 | 2.3079 | 95.0412 | 2.256 | 0.024 | not significant |
| PC 6 | 0.3856 | 2.0295 | 97.0707 | 0.462 | 0.644 | not significant |
| PC 7 | 0.2827 | 1.4880 | 98.5587 | 0.359 | 0.720 | not significant |
| PC 8 | 0.1050 | 0.5525 | 99.1112 | 1.128 | 0.259 | not significant |
| PC 9 | 0.0791 | 0.4162 | 99.5274 | 0.974 | 0.330 | not significant |
| PC 10 | 0.0427 | 0.2248 | 99.7522 | 0.359 | 0.720 | not significant |
| PC 11 | 0.0254 | 0.1338 | 99.8860 | 1.231 | 0.218 | not significant |
| PC 12 | 0.0097 | 0.0508 | 99.9368 | 0.462 | 0.644 | not significant |
| PC 13 | 0.0062 | 0.0324 | 99.9692 | 0.103 | 0.918 | not significant |
| PC 14 | 0.0041 | 0.0218 | 99.9910 | 0.974 | 0.330 | not significant |
| PC 15 | 0.0012 | 0.0063 | 99.9974 | 0.513 | 0.608 | not significant |
| PC 16 | 0.0003 | 0.0014 | 99.9988 | 0.462 | 0.644 | not significant |
| PC 17 | 0.0002 | 0.0011 | 99.9999 | 0.051 | 0.959 | not significant |
| PC 18 | 0.0000 | 0.0001 | 100.0000 | 0.000 | 1.000 | not significant |
| PC 19 | 0.0000 | 0.0000 | 100.0000 | 0.308 | 0.758 | not significant |

Table S10. Eigenvectors from PCA of 19 areal texture parameters that differ between MIS 5e and MIS 7a–c.

| ISO texture parameter | PC 1 | PC 2 | PC 3 | PC 4 | PC 5 | PC 6 | PC 7 | PC 8 | PC 9 | PC 10 | PC 11 | PC 12 | PC 13 | PC 14 | PC 15 | PC 16 | PC 17 | PC 18 | PC 19 |
| --- | --- | --- | --- | --- | --- | --- | --- | --- | --- | --- | --- | --- | --- | --- | --- | --- | --- | --- | --- |
| Sq | 0.2478 | 0.0389 | 0.2741 | -0.0784 | -0.0800 | 0.1223 | -0.0213 | 0.0112 | 0.2874 | 0.0028 | 0.0265 | 0.2654 | -0.1231 | -0.3747 | 0.5758 | 0.4162 | 0.1230 | -0.0673 | -0.0109 |
| Ssk | -0.1856 | 0.4937 | -0.1375 | 0.2470 | -0.4382 | 0.0351 | -0.1143 | 0.3863 | 0.4563 | 0.0643 | 0.2328 | -0.0762 | -0.0040 | 0.0672 | -0.0944 | -0.0019 | -0.0096 | -0.0228 | -0.0014 |
| Sv | 0.2403 | -0.1694 | -0.0469 | 0.1604 | -0.0300 | -0.4637 | 0.1794 | 0.0397 | 0.0808 | 0.2835 | 0.1632 | -0.0643 | 0.0698 | -0.1116 | 0.0103 | -0.0473 | -0.0587 | 0.0162 | -0.7034 |
| Sa | 0.2413 | 0.2623 | 0.2279 | -0.0349 | -0.0479 | -0.0298 | -0.0517 | -0.0784 | -0.0370 | 0.0547 | -0.0608 | -0.1829 | 0.0247 | 0.2513 | 0.5175 | -0.5891 | -0.2826 | -0.0705 | 0.0524 |
| Smc | 0.2147 | 0.4380 | 0.1835 | -0.0669 | 0.0258 | -0.2869 | -0.0506 | -0.1527 | -0.2154 | -0.1476 | 0.0160 | 0.0274 | 0.0548 | 0.2044 | -0.2285 | 0.4576 | -0.2897 | -0.3938 | -0.0221 |
| Sdq | 0.2209 | 0.1392 | -0.4206 | -0.2554 | 0.2166 | 0.0938 | -0.1135 | 0.1721 | -0.1301 | -0.0740 | 0.1801 | -0.4792 | -0.3525 | -0.3978 | 0.0221 | 0.0112 | -0.1414 | -0.0620 | 0.0004 |
| Sdr | 0.1970 | 0.2377 | -0.4667 | -0.3236 | 0.3463 | 0.0695 | -0.0165 | -0.1024 | 0.2498 | 0.3593 | -0.0033 | 0.3799 | 0.1526 | 0.2732 | 0.0128 | -0.0118 | 0.1026 | 0.0446 | 0.0000 |
| Vv | 0.2156 | 0.4458 | 0.1791 | -0.0433 | -0.0241 | -0.2455 | -0.0100 | -0.1469 | -0.1316 | -0.1350 | -0.0375 | 0.0133 | -0.0377 | -0.2210 | -0.1845 | -0.1711 | 0.5177 | 0.4679 | 0.0247 |
| Vvv | 0.2460 | -0.0769 | 0.2765 | -0.0645 | 0.0415 | 0.2056 | -0.1202 | 0.1082 | 0.1169 | 0.1226 | 0.0696 | 0.0058 | 0.0100 | 0.0459 | -0.2377 | 0.1456 | -0.5262 | 0.6260 | 0.0248 |
| Spc | 0.2291 | -0.1515 | -0.1778 | -0.2205 | -0.4668 | 0.0402 | -0.1619 | 0.3029 | -0.5089 | -0.0765 | 0.3016 | 0.2947 | 0.1950 | 0.0994 | 0.0967 | -0.0197 | 0.0684 | 0.0436 | 0.0008 |
| S10z | 0.2501 | 0.0814 | -0.1206 | 0.0662 | -0.0092 | 0.1983 | 0.3081 | 0.2332 | 0.0359 | -0.1779 | -0.4210 | -0.1878 | 0.6574 | -0.2040 | -0.0173 | 0.0450 | -0.0260 | -0.0316 | -0.0020 |
| S5v | 0.2494 | -0.1198 | -0.0323 | 0.0484 | 0.1295 | -0.2000 | 0.1425 | 0.5138 | 0.1241 | -0.3452 | -0.3284 | 0.1534 | -0.4319 | 0.3466 | -0.0058 | -0.0151 | 0.0736 | 0.0263 | 0.0014 |
| Svc | -0.2156 | 0.2326 | 0.2256 | 0.2007 | 0.4870 | 0.1242 | 0.3359 | 0.3985 | -0.3220 | 0.1307 | 0.3539 | 0.1335 | 0.0627 | -0.0161 | 0.1218 | 0.0036 | 0.0280 | 0.0200 | 0.0006 |
| Shh | 0.2240 | 0.1204 | -0.1411 | 0.2882 | -0.3095 | 0.3724 | 0.4643 | -0.2290 | -0.2345 | 0.2906 | -0.1485 | 0.0798 | -0.3864 | 0.0309 | -0.0952 | 0.0341 | -0.0338 | -0.0203 | -0.0003 |
| Sddx | 0.1931 | 0.0019 | -0.1692 | 0.6788 | 0.2160 | 0.0549 | -0.5921 | -0.0183 | -0.1629 | 0.0380 | -0.1378 | 0.1213 | 0.0448 | -0.0610 | 0.0665 | 0.0248 | 0.0167 | 0.0006 | -0.0002 |
| Sddq | 0.2446 | -0.0967 | -0.1216 | 0.2284 | 0.0960 | 0.1642 | 0.2025 | -0.3183 | 0.2291 | -0.5555 | 0.5368 | 0.0069 | 0.0814 | 0.1574 | 0.0036 | -0.0579 | 0.0203 | 0.0282 | 0.0008 |
| Svk | 0.2449 | -0.0780 | 0.2896 | -0.0791 | 0.0525 | 0.1944 | -0.0991 | 0.1162 | 0.1537 | 0.0561 | 0.0741 | 0.2822 | -0.0373 | -0.2984 | -0.4539 | -0.4166 | 0.0085 | -0.4449 | 0.0017 |
| Sak2 | 0.2431 | -0.1317 | 0.2598 | -0.0431 | 0.0403 | 0.2707 | -0.1435 | 0.0845 | 0.0226 | 0.2472 | 0.0957 | -0.4875 | 0.0413 | 0.3954 | -0.0614 | 0.1859 | 0.4783 | -0.1148 | -0.0699 |
| Svkx | 0.2396 | -0.1988 | -0.0450 | 0.1602 | -0.0249 | -0.4396 | 0.1719 | 0.0502 | 0.0848 | 0.3005 | 0.1751 | -0.0966 | 0.0752 | -0.0846 | -0.0171 | 0.0380 | 0.0027 | -0.0411 | 0.7041 |

Table S11. Kruskal–Wallis tests comparing SSFA texture parameters among MIS 5e, MIS 7a–c, and modern Polish wolves; significance was assessed using the Benjamini–Hochberg procedure (FDR = 0.05).

| SSFA parameter | Χ^2^ | p | Benjamini-Hochberg significance |
| --- | --- | --- | --- |
| Area-scale (one corner) |  |  |  |
| Smooth-rough crossover (SRC) | 1.711 | 0.425 | not significant |
| Fractal complexity (Asfc) | 28.183 | <0.0001 | significant |
| Fractal dimension (Das) | 28.183 | <0.0001 | significant |
| Scale of max complexity (Smfc) | 7.878 | 0.019 | significant |
| HAsfc9 (HAsfc9) | 2.282 | 0.319 | not significant |
| HAsfc81 (HAsfc81) | 4.439 | 0.109 | not significant |
| Heterogeneity of Asfc (HAsfc) | 2.282 | 0.319 | not significant |
| Median of Asfc (MedianAsfc) | 28.280 | <0.0001 | significant |
| Mean of Asfc (MeanAsfc) | 28.643 | <0.0001 | significant |
| Standard deviation of Asfc (StdDevAsfc) | 11.407 | 0.003 | significant |
| Median absolute deviation of Asfc (MadAsfc) | 9.854 | 0.007 | significant |
| Length-scale (rows) |  |  |  |
| Smooth-rough crossover (SRC) | 1.818 | 0.403 | not significant |
| Fractal complexity (Lsfc) | 25.587 | <0.0001 | significant |
| Fractal dimension (Dls) | 25.587 | <0.0001 | significant |
| Scale of max complexity (Smfc) | 1.104 | 0.576 | not significant |
| Length-scale anisotropy (Sfrax) (epLsar) | 1.325 | 0.516 | not significant |
| Length-scale anisotropy (NewEplsar) | 0.977 | 0.614 | not significant |

Table S12. Descriptive statistics of SSFA complexity and anisotropy values from all modern and Pleistocene wolves examined.

|  | n |  | Min. | Max. | Range | Median | Mean | SD |
| --- | --- | --- | --- | --- | --- | --- | --- | --- |
| Poland |  |  |  |  |  |  |  |  |
| m1 | 11 | *Asfc* | 7.202 | 11.800 | 4.598 | 9.522 | 9.405 | 1.508 |
|  |  | *New EpLsar* | 0.0174 | 0.0182 | 0.0008 | 0.0177 | 0.0177 | 0.0002 |
|  |  |  |  |  |  |  |  |  |
| m2 | 22 | *Asfc* | 7.460 | 11.912 | 4.451 | 8.996 | 9.241 | 1.070 |
|  |  | *New EpLsar* | 0.0172 | 0.0185 | 0.0013 | 0.0178 | 0.0179 | 0.0003 |
| MIS 5e |  |  |  |  |  |  |  |  |
| m1 | 6 | *Asfc* | 7.560 | 10.763 | 3.204 | 9.007 | 9.087 | 1.175 |
|  |  | *New EpLsar* | 0.0175 | 0.0187 | 0.0012 | 0.0178 | 0.0180 | 0.0005 |
|  |  |  |  |  |  |  |  |  |
| m2 | 7 | *Asfc* | 7.045 | 10.554 | 3.509 | 8.863 | 8.968 | 1.463 |
|  |  | *New EpLsar* | 0.0173 | 0.0184 | 0.0011 | 0.0177 | 0.0177 | 0.0004 |
| MIS 7a-c |  |  |  |  |  |  |  |  |
| m1 | 6 | *Asfc* | 3.234 | 7.349 | 4.115 | 5.799 | 5.667 | 1.519 |
|  |  | *New EpLsar* | 0.0169 | 0.0181 | 0.0011 | 0.0175 | 0.0175 | 0.0005 |
|  |  |  |  |  |  |  |  |  |
| m2 | 7 | *Asfc* | 4.864 | 7.269 | 2.405 | 5.656 | 5.849 | 0.841 |
|  |  | *New EpLsar* | 0.0171 | 0.0182 | 0.0011 | 0.0179 | 0.0177 | 0.0005 |

S13. Results comparing complexity and anisotropy parameters among MIS 5e, MIS 7a–c, and modern Polish wolves based on Kruskal–Wallis tests and subsequent Steel–Dwass pairwise comparisons.

| Texture parameter | Kruskal-Wallis tests | | |  |
| --- | --- | --- | --- | --- |
|  | ***χ*²** | ***p*** |  | |
| *Asfc* | **28.183** | **<0.0001** |  | |
| *NewEpsLar* | 0.977 | 0.614 |  | |
|  |  |  |  | |
| Texture parameter | **Pairwise comparison** | **Steel-Dwass pairwise tests** | | |
|  |  | ***Z*** | ***p*** | |
| *Asfc* | MIS 5e–MIS 7a-c | **4.205** | **<0.0001** | |
|  | MIS 5e–Modern | 0.748 | 0.735 | |
|  | MIS 7a-c– Modern | **4.947** | **<0.0001** | |
| *NewEpsLar* | MIS 5e–MIS 7a-c | 0.872 | 0.658 | |
|  | MIS 5e– Modern | 0.048 | 0.999 | |
|  | MIS 7a-c– Modern | 0.843 | 0.676 | |
|  | MIS 5e– Modern | 0.270 | 0.961 | |
|  | MIS 7a-c– Modern | 0.939 | 0.616 | |

Table S14. Kruskal–Wallis tests comparing areal texture parameters among MIS 5e, MIS 7a–c, and modern Polish wolves; significance was assessed using the Benjamini–Hochberg procedure (FDR = 0.05).

| ISO parameter | Χ^2^ | p | Benjamini-Hochberg significance |
| --- | --- | --- | --- |
| Sq | 15.68 | <0.0001 | significant |
| Ssk | 5.90 | 0.052 | not significant |
| Sku | 4.89 | 0.087 | not significant |
| Sp | 1.37 | 0.504 | not significant |
| Sv | 12.01 | 0.002 | significant |
| Sz | 7.84 | 0.020 | not significant |
| Sa | 17.82 | 0.007 | significant |
| Smr | 1.25 | 0.472 | not significant |
| Smc | 20.45 | 0.005 | significant |
| Sdc | 16.59 | <0.001 | significant |
| Sal | 2.87 | 0.238 | not significant |
| Str | 0.01 | 0.997 | not significant |
| Std | 6.13 | 0.047 | not significant |
| Ssw | 2.11 | 0.349 | not significant |
| Sdq | 26.38 | <0.0001 | significant |
| Sdr | 28.07 | <0.0001 | significant |
| Vm | 14.52 | <0.001 | significant |
| Vv | 20.95 | <0.0001 | significant |
| Vmp | 14.52 | <0.001 | significant |
| Vmc | 16.06 | <0.001 | significant |
| Vvc | 21.96 | <0.0001 | significant |
| Vvv | 13.14 | <0.001 | significant |
| Spd | 1.72 | 0.423 | not significant |
| Spc | 8.79 | 0.007 | significant |
| S10z | 17.58 | <0.001 | significant |
| S5p | 8.33 | 0.024 | significant |
| S5v | 16.14 | 0.001 | significant |
| Sda | 1.99 | 0.370 | not significant |
| Sha | 2.93 | 0.231 | not significant |
| Sdv | 6.58 | 0.037 | not significant |
| Shv | 2.46 | 0.293 | not significant |
| Svd | 1.54 | 0.462 | not significant |
| Svc | 15.80 | <0.001 | significant |
| Shh | 9.02 | 0.011 | significant |
| Shhx | 1.13 | 0.568 | not significant |
| Shhq | 5.47 | 0.065 | not significant |
| Shax | 0.65 | 0.722 | not significant |
| Shaq | 1.61 | 0.447 | not significant |
| Shvx | 1.19 | 0.552 | not significant |
| Shvq | 1.85 | 0.397 | not significant |
| Sdd | 11.50 | <0.0001 | significant |
| Sddx | 14.91 | <0.001 | significant |
| Sddq | 23.20 | <0.0001 | significant |
| Sdax | 1.30 | 0.523 | not significant |
| Sdaq | 2.36 | 0.307 | not significant |
| Sdvx | 4.81 | 0.090 | not significant |
| Sdvq | 5.78 | 0.056 | not significant |
| Shn | 1.69 | 0.429 | not significant |
| Sdn | 1.33 | 0.515 | not significant |
| Sk | 18.38 | <0.001 | significant |
| Spk | 14.21 | <0.001 | significant |
| Svk | 13.21 | 0.001 | significant |
| Smrk1 | 1.54 | 0.462 | not significant |
| Smrk2 | 1.53 | 0.477 | not significant |
| Spq | 20.19 | <0.0001 | significant |
| Svq | 9.21 | 0.001 | significant |
| Smq | 0.32 | 0.853 | not significant |
| Sak1 | 12.02 | 0.002 | significant |
| Sak2 | 11.03 | 0.004 | significant |
| Spkx | 0.87 | 0.648 | not significant |
| Svkx | 11.16 | 0.004 | significant |
| Shrn | 2.36 | 0.307 | not significant |
| Shrnx | 0.87 | 0.649 | not significant |
| Shrnq | 1.48 | 0.477 | not significant |
| Shff | 6.99 | 0.030 | not significant |
| Shffx | 1.03 | 0.598 | not significant |
| Shffq | 1.75 | 0.416 | not significant |
| Shed | 4.69 | 0.096 | not significant |
| Shedx | 0.65 | 0.722 | not significant |
| Shedq | 1.82 | 0.402 | not significant |
| Shar | 0.68 | 0.711 | not significant |
| Sharx | 0.57 | 0.750 | not significant |
| Sharq | 0.36 | 0.837 | not significant |
| Sdrn | 0.67 | 0.714 | not significant |
| Sdrnx | 3.33 | 0.189 | not significant |
| Sdrnq | 3.50 | 0.174 | not significant |
| Sdff | 0.11 | 0.945 | not significant |
| Sdffx | 3.76 | 0.153 | not significant |
| Sdffq | 3.41 | 0.182 | not significant |
| Sded | 1.66 | 0.437 | not significant |
| Sdedx | 1.30 | 0.523 | not significant |
| Sdedq | 2.25 | 0.325 | not significant |
| Sdar | 0.37 | 0.831 | not significant |
| Sdarx | 1.63 | 0.444 | not significant |
| Sdarq | 0.52 | 0.771 | not significant |

Table S15. Eigenvalues from PCA of 30 areal texture parameters that differ significantly between MIS 5e, MIS 7a–c, and modern Polish wolves following application of the Benjamini–Hochberg procedure (FDR = 0.05).

| PC axis | Eigenvalue | Percent variance explained (%) | Cumulative percent (%) | *χ*^2^ | *p* | Benjamini-Hochberg significance |
| --- | --- | --- | --- | --- | --- | --- |
| PC 1 | 18.6534 | 62.1778 | 62.1778 | 19.649 | 0.0001 | significant |
| PC 2 | 3.4811 | 11.6038 | 73.7816 | 4.228 | 0.1208 | not significant |
| PC 3 | 2.8075 | 9.3584 | 83.1400 | 5.559 | 0.0621 | not significant |
| PC 4 | 1.5872 | 5.2908 | 88.4308 | 6.273 | 0.0434 | not significant |
| PC 5 | 1.1717 | 3.9057 | 92.3365 | 6.006 | 0.0496 | not significant |
| PC 6 | 0.7482 | 2.4940 | 94.8305 | 1.880 | 0.3906 | not significant |
| PC 7 | 0.5155 | 1.7182 | 96.5487 | 0.297 | 0.8619 | not significant |
| PC 8 | 0.3598 | 1.1995 | 97.7481 | 8.296 | 0.0158 | not significant |
| PC 9 | 0.2104 | 0.7013 | 98.4494 | 8.325 | 0.0156 | not significant |
| PC 10 | 0.1380 | 0.4600 | 98.9094 | 0.669 | 0.7156 | not significant |
| PC 11 | 0.1153 | 0.3844 | 99.2938 | 1.349 | 0.5094 | not significant |
| PC 12 | 0.0838 | 0.2794 | 99.5732 | 0.593 | 0.7434 | not significant |
| PC 13 | 0.0380 | 0.1266 | 99.6998 | 3.022 | 0.2207 | not significant |
| PC 14 | 0.0292 | 0.0973 | 99.7971 | 1.565 | 0.4573 | not significant |
| PC 15 | 0.0281 | 0.0937 | 99.8907 | 0.528 | 0.7679 | not significant |
| PC 16 | 0.0131 | 0.0435 | 99.9343 | 2.139 | 0.3432 | not significant |
| PC 17 | 0.0104 | 0.0348 | 99.9691 | 0.035 | 0.9824 | not significant |
| PC 18 | 0.0043 | 0.0144 | 99.9834 | 1.716 | 0.4240 | not significant |
| PC 19 | 0.0025 | 0.0082 | 99.9917 | 0.459 | 0.7950 | not significant |
| PC 20 | 0.0013 | 0.0044 | 99.9961 | 0.118 | 0.9425 | not significant |
| PC 21 | 0.0008 | 0.0028 | 99.9989 | 0.323 | 0.8508 | not significant |
| PC 22 | 0.0002 | 0.0008 | 99.9996 | 1.523 | 0.4670 | not significant |
| PC 23 | 0.0001 | 0.0003 | 99.9999 | 2.178 | 0.3365 | not significant |
| PC 24 | 0.0000 | 0.0001 | 100.0000 | 1.102 | 0.5763 | not significant |
| PC 25 | 0.0000 | 0.0000 | 100.0000 | 0.413 | 0.8133 | not significant |
| PC 26 | 0.0000 | 0.0000 | 100.0000 | 0.057 | 0.9721 | not significant |

Table S16. Eigenvectors from PCA of 30 areal texture parameters that differ between MIS 5e, MIS 7a–c, and modern Polish wolves.

| ISO texture parameter | | | | | | | | | | | | | |
| --- | --- | --- | --- | --- | --- | --- | --- | --- | --- | --- | --- | --- | --- |
| Sq | PC 1 | PC 2 | PC 3 | PC 4 | PC 5 | PC 6 | PC 7 | PC 8 | PC 9 | PC 10 | PC 11 | PC 12 | PC 13 |
|  | 0.2146 | -0.1234 | 0.0741 | 0.1010 | -0.1849 | 0.0854 | -0.0756 | 0.0332 | -0.0132 | 0.0856 | 0.1071 | -0.1101 | -0.2213 |
|  | PC 14 | PC 15 | PC 16 | PC 17 | PC 18 | PC 19 | PC 20 | PC 21 | PC 22 | PC 23 | PC 24 | PC 25 | PC 26 |
|  | -0.0160 | 0.1667 | 0.1698 | -0.0214 | -0.0945 | -0.4912 | -0.2869 | 0.1646 | -0.6038 | 0.0543 | -0.0333 | 0.0949 | 0.0057 |
| Sv | PC 1 | PC 2 | PC 3 | PC 4 | PC 5 | PC 6 | PC 7 | PC 8 | PC 9 | PC 10 | PC 11 | PC 12 | PC 13 |
|  | 0.1839 | -0.1737 | -0.1552 | 0.2363 | 0.2419 | -0.0996 | 0.1691 | 0.0478 | 0.1537 | -0.1252 | -0.2142 | -0.0218 | 0.0224 |
|  | PC 14 | PC 15 | PC 16 | PC 17 | PC 18 | PC 19 | PC 20 | PC 21 | PC 22 | PC 23 | PC 24 | PC 25 | PC 26 |
|  | 0.2889 | 0.2165 | -0.1051 | 0.0388 | 0.1494 | -0.0551 | -0.0383 | -0.0129 | 0.0072 | 0.0184 | 0.0015 | 0.0230 | -0.7070 |
| Sa | PC 1 | PC 2 | PC 3 | PC 4 | PC 5 | PC 6 | PC 7 | PC 8 | PC 9 | PC 10 | PC 11 | PC 12 | PC 13 |
|  | 0.2143 | -0.0699 | 0.1862 | 0.0243 | -0.1366 | 0.0687 | -0.0530 | -0.0419 | 0.0288 | -0.0210 | -0.0011 | -0.0003 | 0.0460 |
|  | PC 14 | PC 15 | PC 16 | PC 17 | PC 18 | PC 19 | PC 20 | PC 21 | PC 22 | PC 23 | PC 24 | PC 25 | PC 26 |
|  | -0.0180 | 0.0148 | 0.0606 | -0.0626 | -0.0507 | -0.2330 | -0.2254 | 0.0974 | 0.5279 | 0.1542 | -0.6483 | -0.1853 | -0.0154 |
| Smc | PC 1 | PC 2 | PC 3 | PC 4 | PC 5 | PC 6 | PC 7 | PC 8 | PC 9 | PC 10 | PC 11 | PC 12 | PC 13 |
|  | 0.2111 | 0.0044 | 0.2391 | 0.0197 | 0.0252 | -0.0616 | -0.0216 | -0.0645 | 0.0059 | 0.0741 | 0.0366 | -0.0362 | -0.1293 |
|  | PC 14 | PC 15 | PC 16 | PC 17 | PC 18 | PC 19 | PC 20 | PC 21 | PC 22 | PC 23 | PC 24 | PC 25 | PC 26 |
|  | 0.0975 | -0.0488 | 0.1370 | 0.1274 | 0.0417 | 0.0418 | -0.0324 | -0.1823 | 0.0528 | -0.1861 | 0.1310 | -0.1700 | 0.0022 |
| Sdc | PC 1 | PC 2 | PC 3 | PC 4 | PC 5 | PC 6 | PC 7 | PC 8 | PC 9 | PC 10 | PC 11 | PC 12 | PC 13 |
|  | 0.2032 | -0.0185 | 0.2659 | -0.0286 | -0.0764 | 0.0349 | -0.0100 | -0.1111 | 0.0251 | -0.1214 | -0.1977 | 0.1524 | 0.3603 |
|  | PC 14 | PC 15 | PC 16 | PC 17 | PC 18 | PC 19 | PC 20 | PC 21 | PC 22 | PC 23 | PC 24 | PC 25 | PC 26 |
|  | 0.0725 | -0.2396 | -0.0486 | -0.0394 | 0.0989 | 0.5136 | -0.3593 | 0.0815 | -0.3770 | 0.0782 | -0.1714 | 0.0792 | 0.0026 |
| Sdq | PC 1 | PC 2 | PC 3 | PC 4 | PC 5 | PC 6 | PC 7 | PC 8 | PC 9 | PC 10 | PC 11 | PC 12 | PC 13 |
|  | 0.1917 | -0.0032 | -0.0647 | -0.3347 | 0.1366 | -0.0754 | -0.2289 | 0.4157 | 0.0543 | 0.0109 | -0.0275 | 0.1849 | 0.0759 |
|  | PC 14 | PC 15 | PC 16 | PC 17 | PC 18 | PC 19 | PC 20 | PC 21 | PC 22 | PC 23 | PC 24 | PC 25 | PC 26 |
|  | -0.0761 | 0.0343 | -0.2063 | 0.6618 | -0.2363 | -0.0392 | -0.0446 | 0.0624 | -0.0184 | 0.0341 | -0.0373 | 0.0096 | 0.0003 |
| Sdr | PC 1 | PC 2 | PC 3 | PC 4 | PC 5 | PC 6 | PC 7 | PC 8 | PC 9 | PC 10 | PC 11 | PC 12 | PC 13 |
|  | 0.1870 | 0.0339 | 0.0403 | -0.3318 | 0.1533 | -0.0637 | -0.1841 | 0.5014 | -0.2287 | 0.0049 | -0.2591 | 0.1789 | -0.1397 |
|  | PC 14 | PC 15 | PC 16 | PC 17 | PC 18 | PC 19 | PC 20 | PC 21 | PC 22 | PC 23 | PC 24 | PC 25 | PC 26 |
|  | 0.0098 | 0.1065 | 0.1877 | -0.5404 | 0.1597 | 0.0197 | 0.0259 | -0.0397 | 0.0137 | -0.0244 | 0.0263 | -0.0091 | -0.0004 |
| Vm | PC 1 | PC 2 | PC 3 | PC 4 | PC 5 | PC 6 | PC 7 | PC 8 | PC 9 | PC 10 | PC 11 | PC 12 | PC 13 |
|  | 0.1501 | 0.3583 | -0.1392 | 0.1514 | -0.1736 | 0.0043 | -0.0518 | 0.0533 | 0.0319 | -0.1637 | 0.0036 | 0.0235 | 0.0572 |
|  | PC 14 | PC 15 | PC 16 | PC 17 | PC 18 | PC 19 | PC 20 | PC 21 | PC 22 | PC 23 | PC 24 | PC 25 | PC 26 |
|  | -0.0431 | 0.0996 | -0.0073 | -0.0082 | 0.0557 | 0.0340 | 0.0576 | 0.3467 | 0.0226 | -0.3068 | 0.0080 | -0.0640 | 0.0027 |
| Vv | PC 1 | PC 2 | PC 3 | PC 4 | PC 5 | PC 6 | PC 7 | PC 8 | PC 9 | PC 10 | PC 11 | PC 12 | PC 13 |
|  | 0.2139 | 0.0302 | 0.2198 | 0.0299 | 0.0117 | -0.0589 | -0.0246 | -0.0581 | 0.0080 | 0.0594 | 0.0355 | -0.0331 | -0.1202 |
|  | PC 14 | PC 15 | PC 16 | PC 17 | PC 18 | PC 19 | PC 20 | PC 21 | PC 22 | PC 23 | PC 24 | PC 25 | PC 26 |
|  | 0.0906 | -0.0397 | 0.1312 | 0.1220 | 0.0441 | 0.0426 | -0.0269 | -0.1502 | 0.0524 | -0.2013 | 0.1265 | -0.1681 | 0.0025 |
| Vmp | PC 1 | PC 2 | PC 3 | PC 4 | PC 5 | PC 6 | PC 7 | PC 8 | PC 9 | PC 10 | PC 11 | PC 12 | PC 13 |
|  | 0.1501 | 0.3583 | -0.1392 | 0.1514 | -0.1736 | 0.0043 | -0.0518 | 0.0533 | 0.0319 | -0.1637 | 0.0036 | 0.0235 | 0.0572 |
|  | PC 14 | PC 15 | PC 16 | PC 17 | PC 18 | PC 19 | PC 20 | PC 21 | PC 22 | PC 23 | PC 24 | PC 25 | PC 26 |
|  | -0.0431 | 0.0996 | -0.0073 | -0.0082 | 0.0557 | 0.0340 | 0.0576 | 0.3467 | 0.0226 | -0.3068 | 0.0080 | -0.0640 | 0.0027 |
| Vmc | PC 1 | PC 2 | PC 3 | PC 4 | PC 5 | PC 6 | PC 7 | PC 8 | PC 9 | PC 10 | PC 11 | PC 12 | PC 13 |
|  | 0.1940 | 0.0114 | 0.3031 | -0.0828 | -0.0176 | -0.0601 | 0.0542 | -0.1500 | -0.0242 | -0.2067 | -0.1225 | 0.1196 | 0.3704 |
|  | PC 14 | PC 15 | PC 16 | PC 17 | PC 18 | PC 19 | PC 20 | PC 21 | PC 22 | PC 23 | PC 24 | PC 25 | PC 26 |
|  | -0.1144 | -0.1337 | -0.0386 | -0.0026 | 0.1307 | -0.4148 | 0.5461 | 0.0426 | -0.1237 | 0.2165 | 0.1289 | -0.1537 | -0.0176 |
| Vvc | PC 1 | PC 2 | PC 3 | PC 4 | PC 5 | PC 6 | PC 7 | PC 8 | PC 9 | PC 10 | PC 11 | PC 12 | PC 13 |
|  | 0.1895 | 0.1540 | 0.2575 | -0.0011 | 0.1496 | -0.1912 | 0.0244 | -0.1136 | -0.0234 | 0.0538 | 0.0370 | -0.0315 | -0.1022 |
|  | PC 14 | PC 15 | PC 16 | PC 17 | PC 18 | PC 19 | PC 20 | PC 21 | PC 22 | PC 23 | PC 24 | PC 25 | PC 26 |
|  | 0.1035 | -0.0835 | 0.2441 | 0.1819 | 0.0913 | -0.0415 | 0.0175 | -0.2488 | 0.0044 | -0.3813 | -0.0799 | 0.0724 | 0.0116 |
| Vvv | PC 1 | PC 2 | PC 3 | PC 4 | PC 5 | PC 6 | PC 7 | PC 8 | PC 9 | PC 10 | PC 11 | PC 12 | PC 13 |
|  | 0.1966 | -0.2018 | 0.0887 | 0.0774 | -0.2405 | 0.1966 | -0.1058 | 0.0585 | 0.0624 | 0.0525 | 0.0225 | -0.0264 | -0.1181 |
|  | PC 14 | PC 15 | PC 16 | PC 17 | PC 18 | PC 19 | PC 20 | PC 21 | PC 22 | PC 23 | PC 24 | PC 25 | PC 26 |
|  | 0.0413 | 0.0508 | -0.1099 | -0.0212 | -0.0536 | 0.1822 | -0.0994 | 0.0708 | 0.1241 | 0.1812 | 0.4627 | -0.5537 | -0.0146 |
| Spc | PC 1 | PC 2 | PC 3 | PC 4 | PC 5 | PC 6 | PC 7 | PC 8 | PC 9 | PC 10 | PC 11 | PC 12 | PC 13 |
|  | 0.1506 | -0.1185 | -0.2941 | -0.2618 | -0.0529 | -0.2271 | -0.2656 | -0.3173 | 0.3610 | 0.1893 | 0.3777 | 0.3264 | 0.2213 |
|  | PC 14 | PC 15 | PC 16 | PC 17 | PC 18 | PC 19 | PC 20 | PC 21 | PC 22 | PC 23 | PC 24 | PC 25 | PC 26 |
|  | 0.0975 | 0.2242 | 0.1409 | -0.1596 | 0.0991 | 0.0211 | 0.0108 | -0.0476 | -0.0058 | -0.0068 | 0.0224 | -0.0057 | -0.0002 |
| S10z | PC 1 | PC 2 | PC 3 | PC 4 | PC 5 | PC 6 | PC 7 | PC 8 | PC 9 | PC 10 | PC 11 | PC 12 | PC 13 |
|  | 0.2127 | -0.0684 | -0.1387 | -0.0581 | 0.0026 | -0.0154 | 0.2872 | 0.2330 | 0.1359 | 0.0663 | 0.1881 | -0.2808 | 0.1729 |
|  | PC 14 | PC 15 | PC 16 | PC 17 | PC 18 | PC 19 | PC 20 | PC 21 | PC 22 | PC 23 | PC 24 | PC 25 | PC 26 |
|  | -0.1387 | -0.1918 | 0.1191 | -0.0691 | -0.0186 | 0.0444 | 0.0217 | -0.0144 | 0.0020 | -0.0136 | 0.0027 | -0.0010 | 0.0000 |
| S5p | PC 1 | PC 2 | PC 3 | PC 4 | PC 5 | PC 6 | PC 7 | PC 8 | PC 9 | PC 10 | PC 11 | PC 12 | PC 13 |
|  | 0.1324 | 0.1767 | -0.1242 | -0.4623 | -0.2588 | 0.0909 | 0.3553 | 0.0090 | -0.0447 | 0.2889 | -0.0845 | -0.4240 | 0.1879 |
|  | PC 14 | PC 15 | PC 16 | PC 17 | PC 18 | PC 19 | PC 20 | PC 21 | PC 22 | PC 23 | PC 24 | PC 25 | PC 26 |
|  | 0.2975 | 0.0671 | -0.0615 | 0.0228 | 0.0620 | -0.0301 | 0.0058 | -0.0098 | 0.0050 | 0.0100 | 0.0021 | 0.0026 | -0.0001 |
| S5v | PC 1 | PC 2 | PC 3 | PC 4 | PC 5 | PC 6 | PC 7 | PC 8 | PC 9 | PC 10 | PC 11 | PC 12 | PC 13 |
|  | 0.1927 | -0.1863 | -0.1042 | 0.1889 | 0.1500 | -0.0709 | 0.1602 | 0.2882 | 0.1964 | -0.0803 | 0.2846 | -0.1131 | 0.1111 |
|  | PC 14 | PC 15 | PC 16 | PC 17 | PC 18 | PC 19 | PC 20 | PC 21 | PC 22 | PC 23 | PC 24 | PC 25 | PC 26 |
|  | -0.3433 | -0.2795 | 0.1848 | -0.0999 | -0.0585 | 0.0730 | 0.0241 | -0.0126 | -0.0003 | -0.0228 | 0.0022 | -0.0027 | 0.0001 |
| Svc | PC 1 | PC 2 | PC 3 | PC 4 | PC 5 | PC 6 | PC 7 | PC 8 | PC 9 | PC 10 | PC 11 | PC 12 | PC 13 |
|  | -0.1430 | 0.1562 | 0.3094 | 0.1904 | 0.0455 | 0.2461 | 0.4169 | 0.2640 | 0.0688 | 0.3640 | 0.1896 | 0.4559 | 0.1930 |
|  | PC 14 | PC 15 | PC 16 | PC 17 | PC 18 | PC 19 | PC 20 | PC 21 | PC 22 | PC 23 | PC 24 | PC 25 | PC 26 |
|  | 0.0406 | 0.3043 | 0.0604 | 0.0138 | -0.0205 | 0.0045 | 0.0088 | 0.0032 | -0.0052 | -0.0006 | 0.0060 | -0.0042 | -0.0001 |
| Shh | PC 1 | PC 2 | PC 3 | PC 4 | PC 5 | PC 6 | PC 7 | PC 8 | PC 9 | PC 10 | PC 11 | PC 12 | PC 13 |
|  | 0.1897 | -0.0106 | -0.1966 | -0.2003 | -0.0864 | 0.0690 | 0.4248 | -0.2333 | 0.0893 | 0.0362 | -0.2179 | 0.3534 | -0.4382 |
|  | PC 14 | PC 15 | PC 16 | PC 17 | PC 18 | PC 19 | PC 20 | PC 21 | PC 22 | PC 23 | PC 24 | PC 25 | PC 26 |
|  | -0.4213 | -0.0958 | -0.0752 | 0.0901 | 0.2404 | -0.0054 | -0.0761 | 0.0088 | 0.0107 | 0.0064 | -0.0094 | 0.0069 | -0.0007 |
| Sdd | PC 1 | PC 2 | PC 3 | PC 4 | PC 5 | PC 6 | PC 7 | PC 8 | PC 9 | PC 10 | PC 11 | PC 12 | PC 13 |
|  | 0.2072 | -0.0796 | -0.1609 | -0.0785 | 0.0953 | 0.0936 | 0.2466 | -0.1988 | -0.2820 | -0.1998 | 0.0249 | 0.2271 | -0.0578 |
|  | PC 14 | PC 15 | PC 16 | PC 17 | PC 18 | PC 19 | PC 20 | PC 21 | PC 22 | PC 23 | PC 24 | PC 25 | PC 26 |
|  | 0.2624 | -0.0447 | 0.2024 | -0.0853 | -0.6858 | 0.0848 | 0.1372 | 0.0892 | -0.0067 | -0.0418 | -0.0028 | -0.0166 | 0.0018 |
| Sddx | PC 1 | PC 2 | PC 3 | PC 4 | PC 5 | PC 6 | PC 7 | PC 8 | PC 9 | PC 10 | PC 11 | PC 12 | PC 13 |
|  | 0.1394 | 0.1614 | -0.0684 | 0.0337 | 0.4545 | 0.5170 | -0.2497 | -0.2184 | 0.2412 | 0.3165 | -0.3039 | -0.1658 | 0.0948 |
|  | PC 14 | PC 15 | PC 16 | PC 17 | PC 18 | PC 19 | PC 20 | PC 21 | PC 22 | PC 23 | PC 24 | PC 25 | PC 26 |
|  | -0.1933 | 0.0164 | 0.1033 | -0.0539 | -0.1404 | 0.0012 | 0.0672 | 0.0344 | -0.0162 | -0.0262 | 0.0064 | -0.0042 | 0.0005 |
| Sddq | PC 1 | PC 2 | PC 3 | PC 4 | PC 5 | PC 6 | PC 7 | PC 8 | PC 9 | PC 10 | PC 11 | PC 12 | PC 13 |
|  | 0.1865 | 0.0372 | -0.1032 | -0.0632 | 0.2995 | 0.4059 | -0.0329 | -0.0827 | -0.4562 | -0.1932 | 0.4967 | -0.0018 | 0.0389 |
|  | PC 14 | PC 15 | PC 16 | PC 17 | PC 18 | PC 19 | PC 20 | PC 21 | PC 22 | PC 23 | PC 24 | PC 25 | PC 26 |
|  | 0.0349 | 0.0220 | -0.2040 | 0.0498 | 0.3620 | -0.0278 | -0.1069 | -0.0543 | 0.0092 | 0.0326 | 0.0013 | 0.0063 | -0.0008 |
| Sk | PC 1 | PC 2 | PC 3 | PC 4 | PC 5 | PC 6 | PC 7 | PC 8 | PC 9 | PC 10 | PC 11 | PC 12 | PC 13 |
|  | 0.1787 | 0.1182 | 0.3007 | -0.0745 | 0.1398 | -0.2379 | 0.0838 | -0.1491 | -0.0126 | -0.1278 | 0.0885 | -0.2365 | -0.0781 |
|  | PC 14 | PC 15 | PC 16 | PC 17 | PC 18 | PC 19 | PC 20 | PC 21 | PC 22 | PC 23 | PC 24 | PC 25 | PC 26 |
|  | -0.2465 | 0.4687 | 0.0667 | 0.0138 | -0.0809 | 0.2166 | -0.0443 | 0.1992 | 0.1751 | 0.3251 | 0.1966 | 0.3314 | 0.0356 |
| Spk | PC 1 | PC 2 | PC 3 | PC 4 | PC 5 | PC 6 | PC 7 | PC 8 | PC 9 | PC 10 | PC 11 | PC 12 | PC 13 |
|  | 0.1537 | 0.3411 | -0.1452 | 0.1396 | -0.2106 | 0.0283 | -0.0233 | 0.0336 | 0.0238 | -0.2160 | -0.0581 | 0.0149 | 0.1236 |
|  | PC 14 | PC 15 | PC 16 | PC 17 | PC 18 | PC 19 | PC 20 | PC 21 | PC 22 | PC 23 | PC 24 | PC 25 | PC 26 |
|  | -0.1930 | 0.2126 | -0.1493 | -0.0965 | -0.2197 | -0.0420 | -0.1170 | -0.7160 | -0.0653 | 0.1053 | -0.0088 | -0.0235 | 0.0004 |
| Svk | PC 1 | PC 2 | PC 3 | PC 4 | PC 5 | PC 6 | PC 7 | PC 8 | PC 9 | PC 10 | PC 11 | PC 12 | PC 13 |
|  | 0.1993 | -0.1951 | 0.0666 | 0.0927 | -0.2270 | 0.1781 | -0.1121 | 0.0813 | 0.0506 | 0.0843 | 0.0953 | -0.0771 | -0.2713 |
|  | PC 14 | PC 15 | PC 16 | PC 17 | PC 18 | PC 19 | PC 20 | PC 21 | PC 22 | PC 23 | PC 24 | PC 25 | PC 26 |
|  | 0.0368 | 0.1676 | -0.1456 | 0.0311 | 0.0454 | 0.3431 | 0.5926 | -0.0932 | -0.1590 | 0.0091 | -0.3660 | 0.1339 | -0.0026 |
| Spq | PC 1 | PC 2 | PC 3 | PC 4 | PC 5 | PC 6 | PC 7 | PC 8 | PC 9 | PC 10 | PC 11 | PC 12 | PC 13 |
|  | 0.1865 | 0.2219 | 0.1050 | 0.0919 | 0.1866 | -0.3003 | 0.0053 | -0.0431 | 0.0262 | 0.3225 | 0.1501 | 0.0242 | -0.1590 |
|  | PC 14 | PC 15 | PC 16 | PC 17 | PC 18 | PC 19 | PC 20 | PC 21 | PC 22 | PC 23 | PC 24 | PC 25 | PC 26 |
|  | 0.0717 | -0.2480 | -0.6394 | -0.2947 | -0.1932 | -0.0764 | -0.0169 | 0.0911 | -0.0389 | 0.0194 | -0.0228 | 0.0018 | 0.0002 |
| Svq | PC 1 | PC 2 | PC 3 | PC 4 | PC 5 | PC 6 | PC 7 | PC 8 | PC 9 | PC 10 | PC 11 | PC 12 | PC 13 |
|  | 0.1544 | -0.1784 | -0.2150 | 0.3028 | -0.0918 | -0.2535 | -0.0617 | -0.0816 | -0.5772 | 0.4559 | -0.1735 | 0.0198 | 0.2545 |
|  | PC 14 | PC 15 | PC 16 | PC 17 | PC 18 | PC 19 | PC 20 | PC 21 | PC 22 | PC 23 | PC 24 | PC 25 | PC 26 |
|  | -0.2381 | 0.0863 | 0.0587 | 0.1111 | 0.0394 | 0.0575 | 0.0185 | 0.0063 | 0.0411 | -0.0120 | 0.0069 | -0.0055 | -0.0006 |
| Sak1 | PC 1 | PC 2 | PC 3 | PC 4 | PC 5 | PC 6 | PC 7 | PC 8 | PC 9 | PC 10 | PC 11 | PC 12 | PC 13 |
|  | 0.1253 | 0.3857 | -0.1793 | 0.2169 | -0.0693 | -0.0083 | -0.1008 | 0.0833 | 0.0174 | 0.1111 | 0.0297 | 0.1122 | -0.1686 |
|  | PC 14 | PC 15 | PC 16 | PC 17 | PC 18 | PC 19 | PC 20 | PC 21 | PC 22 | PC 23 | PC 24 | PC 25 | PC 26 |
|  | 0.2857 | -0.3233 | 0.3165 | 0.1455 | 0.1362 | 0.0253 | 0.0637 | 0.0119 | 0.0701 | 0.5598 | 0.0616 | 0.1640 | -0.0024 |
| Sak2 | PC 1 | PC 2 | PC 3 | PC 4 | PC 5 | PC 6 | PC 7 | PC 8 | PC 9 | PC 10 | PC 11 | PC 12 | PC 13 |
|  | 0.1871 | -0.2218 | 0.0960 | 0.0578 | -0.2774 | 0.2442 | -0.1056 | 0.0403 | 0.0529 | 0.0066 | -0.0626 | 0.1143 | 0.0985 |
|  | PC 14 | PC 15 | PC 16 | PC 17 | PC 18 | PC 19 | PC 20 | PC 21 | PC 22 | PC 23 | PC 24 | PC 25 | PC 26 |
|  | 0.0700 | -0.1626 | -0.1367 | -0.0636 | -0.0356 | -0.1564 | -0.0539 | -0.0380 | 0.3280 | -0.2019 | 0.3146 | 0.6268 | 0.0015 |
| Svkx | PC 1 | PC 2 | PC 3 | PC 4 | PC 5 | PC 6 | PC 7 | PC 8 | PC 9 | PC 10 | PC 11 | PC 12 | PC 13 |
|  | 0.1812 | -0.1889 | -0.1641 | 0.2394 | 0.2266 | -0.0811 | 0.1633 | 0.0546 | 0.1557 | -0.1225 | -0.2218 | -0.0067 | 0.0348 |
|  | PC 14 | PC 15 | PC 16 | PC 17 | PC 18 | PC 19 | PC 20 | PC 21 | PC 22 | PC 23 | PC 24 | PC 25 | PC 26 |
|  | 0.2975 | 0.1919 | -0.1161 | 0.0336 | 0.1566 | -0.0741 | -0.0243 | -0.0175 | 0.0142 | 0.0261 | -0.0103 | -0.0138 | 0.7056 |

Table S17. PCA of 30 areal texture parameters that differ between MIS 5e, MIS 7a–c, and modern Polish wolves; Steel–Dwass pairwise comparisons of PC 1 values.

| ***Z*** (MIS 5e vs MIS 7a-c) | ***p*** (MIS 5e vs MIS 7a-c) | ***Z*** (MIS 5e vs Modern Poland) | ***p*** (MIS 5e vs Modern Poland) | ***Z*** (MIS 7a-c vs Modern Poland) | ***p*** (MIS 7a-c vs Modern Poland) |
| --- | --- | --- | --- | --- | --- |
| 3.128 | 0.0050 | 1.766 | 0.1812 | 4.056 | 0.0001 |

Table S18. Kruskal–Wallis tests* comparing SSFA texture parameters among Polish regions, and Wilcoxon two-sample tests comparing SSFA texture parameters by season (October–March vs. April–September), age, and sex. No parameters remained significant following application of the Benjamini–Hochberg procedure (FDR = 0.05).

|  | Region* |  | Season |  | Age |  | Sex |  |
| --- | --- | --- | --- | --- | --- | --- | --- | --- |
| SSFA parameter | χ2 | p | Z | p | Z | p | Z | p |
| Area-scale (one corner) |  |  |  |  |  |  |  |  |
| Smooth-rough crossover (SRC) | 5.606 | 0.231 | 0.298 | 0.766 | 1.014 | 0.311 | 1.101 | 0.271 |
| Fractal complexity (Asfc) | 1.385 | 0.847 | 0.835 | 0.404 | 0.060 | 0.952 | 0.058 | 0.954 |
| Fractal dimension (Das) | 1.385 | 0.847 | 0.835 | 0.404 | 0.060 | 0.952 | 0.058 | 0.954 |
| Scale of max complexity (Smfc) | 5.564 | 0.234 | 0.000 | 1.000 | 0.090 | 0.929 | 0.232 | 0.816 |
| HAsfc9 (HAsfc9) | 4.793 | 0.309 | 0.298 | 0.766 | 0.358 | 0.721 | 0.811 | 0.417 |
| HAsfc81 (HAsfc81) | 3.222 | 0.521 | 0.239 | 0.811 | 0.298 | 0.766 | 0.174 | 0.862 |
| Heterogeneity of Asfc (HAsfc) | 4.793 | 0.309 | 0.298 | 0.766 | 0.358 | 0.721 | 0.811 | 0.417 |
| Median of Asfc (MedianAsfc) | 0.659 | 0.956 | 0.954 | 0.340 | 0.179 | 0.858 | 0.290 | 0.772 |
| Mean of Asfc (MeanAsfc) | 2.368 | 0.668 | 1.252 | 0.210 | 0.060 | 0.952 | 0.000 | 1.000 |
| Standard deviation of Asfc (StdDevAsfc) | 8.395 | 0.078 | 0.537 | 0.592 | 0.179 | 0.858 | 0.406 | 0.685 |
| Median absolute deviation of Asfc (MadAsfc) | 4.649 | 0.325 | 0.000 | 1.000 | 0.477 | 0.633 | 0.116 | 0.908 |
| Length-scale (rows) |  |  |  |  |  |  |  |  |
| Smooth-rough crossover (SRC) | 3.949 | 0.413 | 0.000 | 1.000 | 0.417 | 0.676 | 0.637 | 0.524 |
| Fractal complexity (Lsfc) | 0.526 | 0.971 | 0.716 | 0.474 | 0.239 | 0.811 | 0.000 | 1.000 |
| Fractal dimension (Dls) | 0.526 | 0.971 | 0.716 | 0.474 | 0.239 | 0.811 | 0.000 | 1.000 |
| Scale of max complexity (Smfc) | 3.527 | 0.474 | 1.730 | 0.084 | 1.193 | 0.233 | 0.985 | 0.325 |
| Length-scale anisotropy (Sfrax) (epLsar) | 10.086 | 0.039 | 1.073 | 0.283 | 0.477 | 0.633 | 1.738 | 0.082 |
| Length-scale anisotropy  (NewEplsar) | 3.816 | 0.432 | 0.656 | 0.512 | 0.119 | 0.905 | 0.811 | 0.417 |

Table S19. Kruskal–Wallis tests* comparing areal texture parameters among Polish regions, and Wilcoxon two-sample tests comparing ISO texture parameters by season (October–March vs. April–September), age, and sex. No parameters remained significant following application of the Benjamini–Hochberg procedure (FDR = 0.05).

|  | Region* |  | Season |  | Age |  | Sex |  |
| --- | --- | --- | --- | --- | --- | --- | --- | --- |
| ISO | χ2 | p | Z | p | Z | p | Z | p |
| Sq | 5.776 | 0.217 | 1.133 | 0.257 | 1.073 | 0.283 | 0.869 | 0.385 |
| Ssk | 3.335 | 0.503 | 0.000 | 1.000 | 1.431 | 0.152 | 1.448 | 0.148 |
| Sku | 7.783 | 0.100 | 0.894 | 0.371 | 1.073 | 0.283 | 1.390 | 0.164 |
| Sp | 2.576 | 0.631 | 0.596 | 0.551 | 0.894 | 0.371 | 1.970 | 0.049 |
| Sv | 10.261 | 0.036 | 0.179 | 0.858 | 1.014 | 0.311 | 0.869 | 0.385 |
| Sz | 6.632 | 0.157 | 0.537 | 0.592 | 0.179 | 0.858 | 0.232 | 0.817 |
| Sa | 3.382 | 0.496 | 1.133 | 0.257 | 1.193 | 0.233 | 0.811 | 0.417 |
| Smr | 2.668 | 0.615 | 0.537 | 0.592 | 0.477 | 0.633 | 1.622 | 0.105 |
| Smc | 3.560 | 0.469 | 1.252 | 0.210 | 0.954 | 0.340 | 0.579 | 0.562 |
| Sdc | 3.486 | 0.480 | 1.312 | 0.190 | 0.894 | 0.371 | 0.521 | 0.602 |
| Sal | 12.425 | 0.014 | 0.596 | 0.551 | 1.729 | 0.084 | 1.970 | 0.049 |
| Str | 6.541 | 0.162 | 1.789 | 0.074 | 0.239 | 0.811 | 0.290 | 0.772 |
| Std | 4.647 | 0.325 | 0.119 | 0.905 | 2.147 | 0.032 | 1.333 | 0.183 |
| Ssw | 3.862 | 0.425 | 0.537 | 0.592 | 1.133 | 0.257 | 1.043 | 0.297 |
| Sdq | 1.736 | 0.784 | 1.431 | 0.152 | 0.239 | 0.811 | 1.159 | 0.247 |
| Sdr | 1.725 | 0.786 | 1.371 | 0.170 | 0.060 | 0.952 | 0.521 | 0.602 |
| Vm | 5.563 | 0.234 | 0.656 | 0.512 | 0.298 | 0.766 | 0.000 | 1.000 |
| Vv | 4.007 | 0.405 | 1.431 | 0.152 | 0.835 | 0.404 | 0.463 | 0.643 |
| Vmp | 5.563 | 0.234 | 0.656 | 0.512 | 0.298 | 0.766 | 0.000 | 1.000 |
| Vmc | 2.821 | 0.588 | 1.073 | 0.283 | 1.252 | 0.210 | 0.637 | 0.524 |
| Vvc | 3.667 | 0.453 | 0.656 | 0.512 | 0.716 | 0.474 | 0.290 | 0.772 |
| Vvv | 5.589 | 0.232 | 1.073 | 0.283 | 1.014 | 0.311 | 0.811 | 0.417 |
| Spd | 8.899 | 0.064 | 0.716 | 0.474 | 0.894 | 0.371 | 0.637 | 0.524 |
| Spc | 2.882 | 0.578 | 1.550 | 0.121 | 0.775 | 0.438 | 1.506 | 0.132 |
| S10z | 10.596 | 0.031 | 0.060 | 0.952 | 0.358 | 0.721 | 0.174 | 0.862 |
| S5p | 2.407 | 0.661 | 0.596 | 0.551 | 1.014 | 0.311 | 1.448 | 0.148 |
| S5v | 8.861 | 0.065 | 0.179 | 0.858 | 1.014 | 0.311 | 1.043 | 0.297 |
| Sda | 4.926 | 0.295 | 0.119 | 0.905 | 0.537 | 0.592 | 0.058 | 0.954 |
| Sha | 6.395 | 0.172 | 1.193 | 0.233 | 0.894 | 0.371 | 0.637 | 0.524 |
| Sdv | 4.712 | 0.318 | 0.417 | 0.676 | 1.491 | 0.136 | 1.159 | 0.247 |
| Shv | 6.016 | 0.198 | 0.239 | 0.811 | 0.656 | 0.512 | 0.058 | 0.954 |
| Svd | 6.863 | 0.143 | 0.060 | 0.952 | 0.358 | 0.721 | 0.116 | 0.908 |
| Svc | 3.113 | 0.539 | 1.312 | 0.190 | 0.656 | 0.512 | 0.637 | 0.524 |
| Shh | 7.969 | 0.093 | 1.193 | 0.233 | 0.060 | 0.952 | 0.927 | 0.354 |
| Shhx | 3.260 | 0.515 | 0.060 | 0.952 | 0.298 | 0.766 | 1.275 | 0.202 |
| Shhq | 5.602 | 0.231 | 1.610 | 0.107 | 0.060 | 0.952 | 1.564 | 0.118 |
| Shax | 4.233 | 0.375 | 0.239 | 0.811 | 0.477 | 0.633 | 0.637 | 0.524 |
| Shaq | 7.290 | 0.121 | 0.000 | 1.000 | 0.358 | 0.721 | 0.000 | 1.000 |
| Shvx | 5.968 | 0.202 | 0.596 | 0.551 | 1.133 | 0.257 | 0.232 | 0.817 |
| Shvq | 6.979 | 0.137 | 0.477 | 0.633 | 0.537 | 0.592 | 0.058 | 0.954 |
| Sdd | 4.695 | 0.320 | 0.119 | 0.905 | 1.014 | 0.311 | 0.058 | 0.954 |
| Sddx | 4.880 | 0.300 | 0.477 | 0.633 | 0.477 | 0.633 | 1.333 | 0.183 |
| Sddq | 4.362 | 0.359 | 0.358 | 0.721 | 0.954 | 0.340 | 0.753 | 0.451 |
| Sdax | 4.303 | 0.367 | 0.358 | 0.721 | 0.596 | 0.551 | 0.869 | 0.385 |
| Sdaq | 8.160 | 0.086 | 0.179 | 0.858 | 0.417 | 0.676 | 0.348 | 0.728 |
| Sdvx | 1.949 | 0.745 | 0.537 | 0.592 | 0.954 | 0.340 | 0.521 | 0.602 |
| Sdvq | 1.465 | 0.833 | 0.358 | 0.721 | 1.312 | 0.190 | 0.927 | 0.354 |
| Shn | 9.438 | 0.051 | 0.924 | 0.355 | 0.954 | 0.340 | 0.695 | 0.487 |
| Sdn | 5.049 | 0.282 | 0.268 | 0.788 | 0.388 | 0.698 | 0.145 | 0.885 |
| Sk | 2.825 | 0.587 | 1.193 | 0.233 | 0.477 | 0.633 | 0.000 | 1.000 |
| Spk | 6.366 | 0.173 | 0.835 | 0.404 | 0.119 | 0.905 | 0.000 | 1.000 |
| Svk | 6.025 | 0.197 | 1.014 | 0.311 | 0.954 | 0.340 | 0.811 | 0.417 |
| Smrk1 | 0.999 | 0.910 | 1.252 | 0.210 | 1.073 | 0.283 | 0.637 | 0.524 |
| Smrk2 | 6.166 | 0.187 | 1.133 | 0.257 | 1.133 | 0.257 | 0.985 | 0.325 |
| Spq | 4.821 | 0.306 | 1.014 | 0.311 | 0.239 | 0.811 | 0.406 | 0.685 |
| Svq | 13.379 | 0.010 | 0.179 | 0.858 | 1.312 | 0.190 | 1.448 | 0.148 |
| Smq | 5.015 | 0.286 | 0.775 | 0.438 | 0.656 | 0.512 | 0.348 | 0.728 |
| Sak1 | 3.305 | 0.508 | 0.000 | 1.000 | 0.775 | 0.438 | 0.348 | 0.728 |
| Sak2 | 5.530 | 0.237 | 0.954 | 0.340 | 1.193 | 0.233 | 1.043 | 0.297 |
| Spkx | 2.406 | 0.662 | 0.298 | 0.766 | 1.014 | 0.311 | 2.028 | 0.043 |
| Svkx | 9.965 | 0.041 | 0.179 | 0.858 | 1.073 | 0.283 | 0.927 | 0.354 |
| Shrn | 7.486 | 0.112 | 0.239 | 0.811 | 0.119 | 0.905 | 0.985 | 0.325 |
| Shrnx | 2.600 | 0.627 | 0.537 | 0.591 | 0.000 | 1.000 | 0.609 | 0.543 |
| Shrnq | 6.425 | 0.170 | 1.073 | 0.283 | 0.656 | 0.512 | 0.463 | 0.643 |
| Shff | 6.419 | 0.170 | 1.789 | 0.074 | 0.656 | 0.512 | 1.333 | 0.183 |
| Shffx | 5.353 | 0.253 | 0.477 | 0.633 | 0.954 | 0.340 | 0.637 | 0.524 |
| Shffq | 4.463 | 0.347 | 0.298 | 0.766 | 1.133 | 0.257 | 1.101 | 0.271 |
| Shed | 3.317 | 0.506 | 1.789 | 0.074 | 0.596 | 0.551 | 0.869 | 0.385 |
| Shedx | 4.154 | 0.386 | 0.239 | 0.811 | 0.537 | 0.592 | 0.695 | 0.487 |
| Shedq | 7.228 | 0.124 | 0.656 | 0.512 | 0.417 | 0.676 | 0.116 | 0.908 |
| Shar | 5.665 | 0.226 | 0.537 | 0.592 | 0.179 | 0.858 | 0.927 | 0.354 |
| Sharx | 7.638 | 0.106 | 0.209 | 0.835 | 0.149 | 0.881 | 0.608 | 0.543 |
| Sharq | 9.943 | 0.041 | 0.596 | 0.551 | 0.716 | 0.474 | 0.000 | 1.000 |
| Sdrn | 3.168 | 0.530 | 0.239 | 0.811 | 1.729 | 0.084 | 0.753 | 0.451 |
| Sdrnx | 3.979 | 0.409 | 1.133 | 0.257 | 1.789 | 0.074 | 1.159 | 0.247 |
| Sdrnq | 6.914 | 0.141 | 0.119 | 0.905 | 1.073 | 0.283 | 0.753 | 0.451 |
| Sdff | 3.562 | 0.469 | 1.014 | 0.311 | 0.656 | 0.512 | 0.406 | 0.685 |
| Sdffx | 1.210 | 0.877 | 0.417 | 0.676 | 2.087 | 0.037 | 0.927 | 0.354 |
| Sdffq | 5.549 | 0.235 | 1.312 | 0.190 | 2.385 | 0.017 | 1.912 | 0.056 |
| Sded | 3.716 | 0.446 | 0.119 | 0.905 | 0.477 | 0.633 | 0.290 | 0.772 |
| Sdedx | 4.316 | 0.365 | 0.358 | 0.721 | 0.596 | 0.551 | 0.869 | 0.385 |
| Sdedq | 8.661 | 0.070 | 0.537 | 0.592 | 0.716 | 0.474 | 0.116 | 0.908 |
| Sdar | 1.549 | 0.818 | 1.193 | 0.233 | 1.729 | 0.084 | 1.217 | 0.224 |
| Sdarx | 3.668 | 0.453 | 1.491 | 0.136 | 1.908 | 0.056 | 0.811 | 0.417 |
| Sdarq | 1.548 | 0.818 | 1.193 | 0.233 | 1.193 | 0.233 | 0.232 | 0.817 |

Supporting Information References

Benjamini, Y. and Hochberg, Y. (1995). Controlling the false discovery rate: a practical and powerful approach to multiple testing. *J. R. Stat. Soc. Ser. B Methodol.*, *57*(1), pp.289-300.

[https://doi-org.proxyiub.uits.iu.edu/ 10.1111/j.2517-6161.1995.tb02031.x](https://doi-org.proxyiub.uits.iu.edu/%2010.1111/j.2517-6161.1995.tb02031.x)

Berger, A., Crucifix, M., Hodell, D.A., Mangili, C., McManus, J.F., Otto-Bliesner, B. et al. (Past Interglacials Working Group of PAGES) (2015). Interglacials of the last 800,000 years. *Rev. Geophys.* 54(1), pp. 162-219.

<https://doi-org.proxyiub.uits.iu.edu/10.1002/2015RG000482>

Bridgland, D.R. (1994). Quaternary of the Thames (Geological Conservation Review Series, no. 7). *Chamman and Hall, London*, UK.

Candy, I. and Schreve, D.C. (2007). Land–sea correlation of Middle Pleistocene temperate sub-stages using high-precision uranium-series dating of tufa deposits from southern England. *Quat. Sci. Rev.*, *26*(9-10), pp.1223-1235.

Catcott, A. (1761). A Treatise on the Deluge. Second edition. Withers and Prince, London, UK. <https://doi-org.proxyiub.uits.iu.edu/10.1016/j.quascirev.2007.01.012>

Currant, A.P. (2004). The Quaternary Mammal Collections at the Somerset County Museum, Taunton. In: *The Quaternary Mammals of Southern and Eastern England. Field Guide*, Schreve, D.C. (Ed.), Quaternary Research Association, London, pp 101-109.

Currant, A. P., and Jacobi, R. (2011). The mammal faunas of the British Late Pleistocene. *Developments in Quaternary Sciences*, *14*, pp. 165-180.

<https://doi-org.proxyiub.uits.iu.edu/10.1016/B978-0-444-53597-9.00010-8>

Davies, W. (1874). *Catalogue of the Pleistocene Vertebrata from the neighbourhood of Ilford, Essex, in the collection of Sir Antonio Brady*. London (printed for private circulation).

Donovan, D.T. (1954). A bibliography of the Palaeolithic and Pleistocene sites of the Mendip, Bath and Bristol area. *Proc. Univ. Bristol Spelaeol. Soc.*, *7*, pp.23-24.

Gipson, P. S., Ballard, W. B., Nowak, R. M., & Mech, L. D. (2000). Accuracy and Precision of Estimating Age of Gray Wolves by Tooth Wear. *J. Wildl. Manag.,* *64*(3), pp. 752–758. <https://doi.org/10.2307/3802745>

Green, H. S. (1984). *Pontnewydd Cave: A Lower Palaeolithic hominid site in Wales: The first report* (National Museum of Wales Quaternary Studies Monographs, No. 1). National Museum of Wales, Cardiff.

Green, H.S. (1995). Pontnewydd Cave, Wales, a later Middle Pleistocene hominid and archaeological site: a review of stratigraphy, dating, taphonomy and interpretation. In *Human Evolution in Europe and the Atapuerca evidence*, Bermúdez de Castro, J.M., Arsuaga, J.L. and Carbonell, E. (Eds.), Junta de Castilla y León., pp.37-55.

Hodge, E., Hoffmann, D., Richards, D. and Smart, P. (2016). Uranium-series ages for speleothem and tufa deposits associated with Quaternary mammalian fossil evidence in England and Wales. *Proceedings of the University of Bristol Spelaeological Society*, *27*(1), pp.73-80.

Huxtable, J. (1984). Thermoluminescence (TL) studies on burnt flint and stones. In *Pontnewydd Cave. A Lower Palaeolithic hominid site in Wales*. Green, H.S. (Ed.) The First Report, National Museum of Wales, Cardiff, pp. 106-107.

Keen, D.H. (2001). Towards a late Middle Pleistocene non-marine molluscan biostratigraphy for the British Isles. *Quaternary Science Reviews*, *20*(16-17), pp.1657-1665. <https://doi-org.proxyiub.uits.iu.edu/10.1016/S0277-3791(01)00030-0>

McDonald, J.H. (2014). Multiple comparisons: Controlling the false discovery rate: Benjamini–Hochberg Procedure. *Handbook of Biological Statistics*. Baltimore, Maryland: Sparkly House Publishing, pp.254-60.

Murton, J.B., Baker, A., Bowen, D.Q., Caseldine, C.J., Coope, G.R., Currant, A.P. et al. (2001). A late Middle Pleistocene temperate-periglacial-temperate sequence (Oxygen Isotope Stages 7-5e) near Marsworth, Buckinghamshire, UK. *Quat. Sci. Rev.*, *20*(18), pp.1787-1825.

[https://doi-org.proxyiub.uits.iu.edu/ 10.1016/S0277-3791(01)00004-X](https://doi-org.proxyiub.uits.iu.edu/%2010.1016/S0277-3791(01)00004-X)

Peel, M. C., Finlayson, B. L., & McMahon, T. A. (2007). Updated world map of the Köppen-Geiger climate classification. *Hydrol. Earth Syst. Sci.*, *11*(5), 1633-1644. <https://doi.org/10.5194/hess-11-1633-2007>

Penkman, K.E., Preece, R.C., Bridgland, D.R., Keen, D.H., Meijer, T., Parfitt, S.A. et al. (2013). An aminostratigraphy for the British Quaternary based on Bithynia opercula. *Quaternary Science Reviews*, *61*, pp.111-134.

<https://doi-org.proxyiub.uits.iu.edu/10.1016/j.quascirev.2012.10.046>

Schreve, D.C. (1998). *Mammalian biostratigraphy of the later Middle Pleistocene in Britain*. University of London, University College London, UK.

Schreve, D.C. (2001). Differentiation of the British late Middle Pleistocene interglacials: the evidence from mammalian biostratigraphy. *Quat. Sci. Rev.*, *20*(16-17), pp.1693-1705.

<https://doi-org.proxyiub.uits.iu.edu/10.1016/S0277-3791(01)00033-6>

Schwarcz, H. P. (1984). *Uranium-series dating and stable isotope analyses of calcite deposits*. In *Studies of Pontnewydd Cave, Wales*, H. S. Green (Ed.), National Museum of Wales, pp.88-97.

Sutcliffe, A.J. (1958). J*oint Mitnor Cave, Buckfastleigh: A Report on Excavations Carried Out During 1939-41 by the Late AH Ogilvie*. Torquay Natural History Society.

Wilson, E. (1885). The Bone-Cave or Fissure of Durdham Down. PBNS New Series 5, pp. 31-45.
